# Supplementary figures and images for: Effects of a six-week weighted-implement throwing program on baseball pitching velocity, kinematics, arm stress, and arm range of motion
Source: PeerJ. 2018 Nov 23;6:e6003. doi: 10.7717/peerj.6003 (PMC6254244; doi:10.7717/peerj.6003)

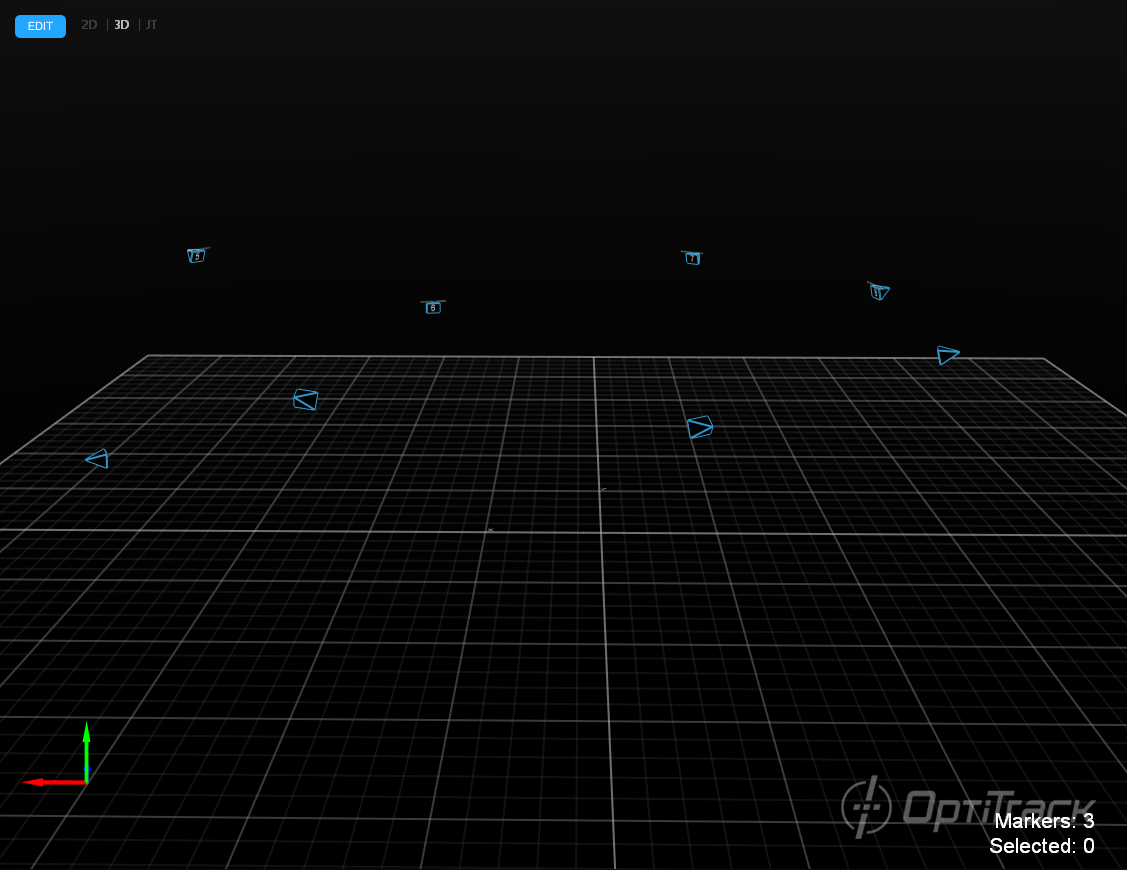

Supplement: Supplemental Information 1 — The 8 camera montage as seen from Optitrack’s Motive software. [file peerj-06-6003-s001.png]

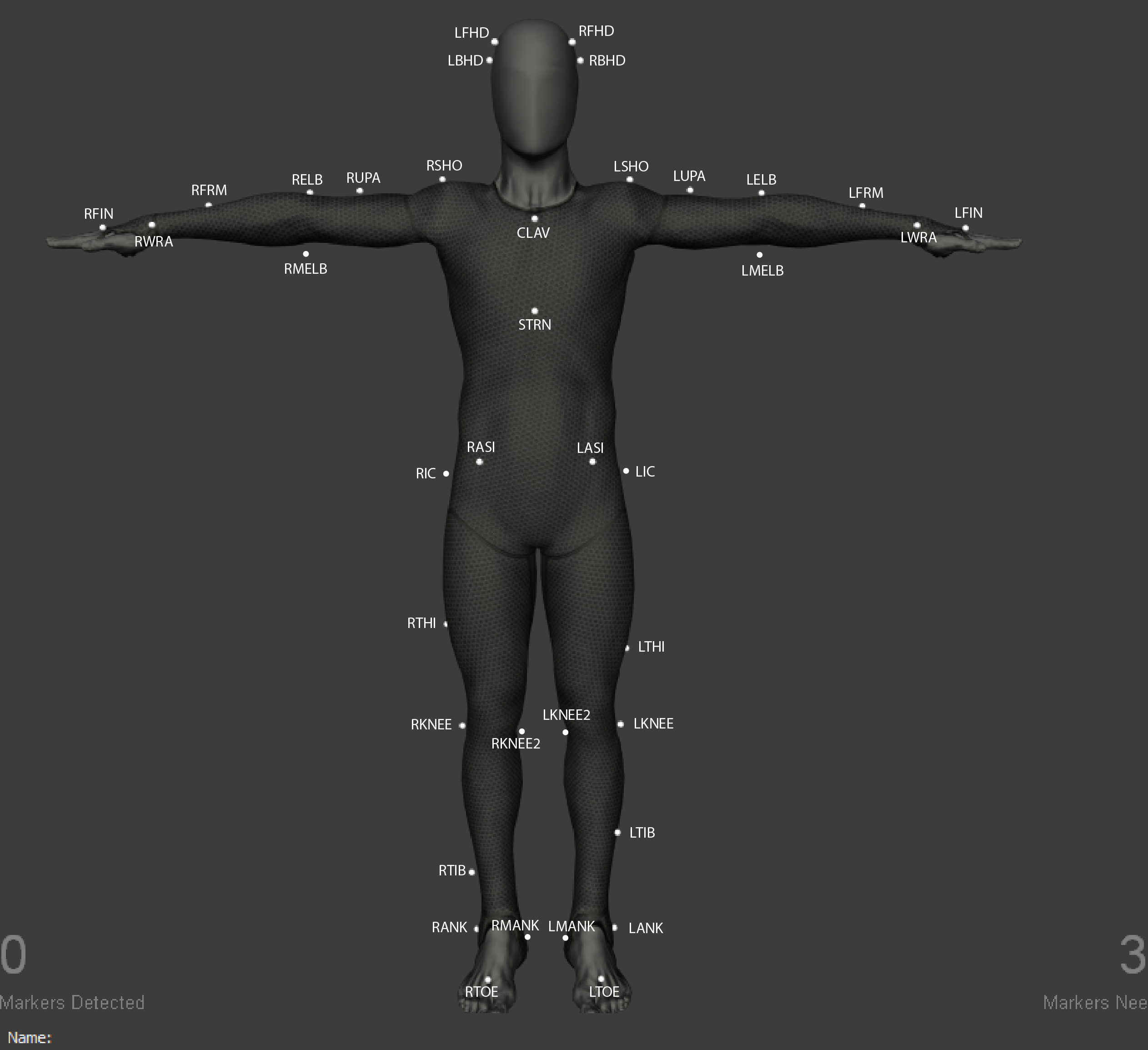

Supplement: Supplemental Information 2 — The front anatomical view of how body markers were placed on subjects. [file peerj-06-6003-s002.png]

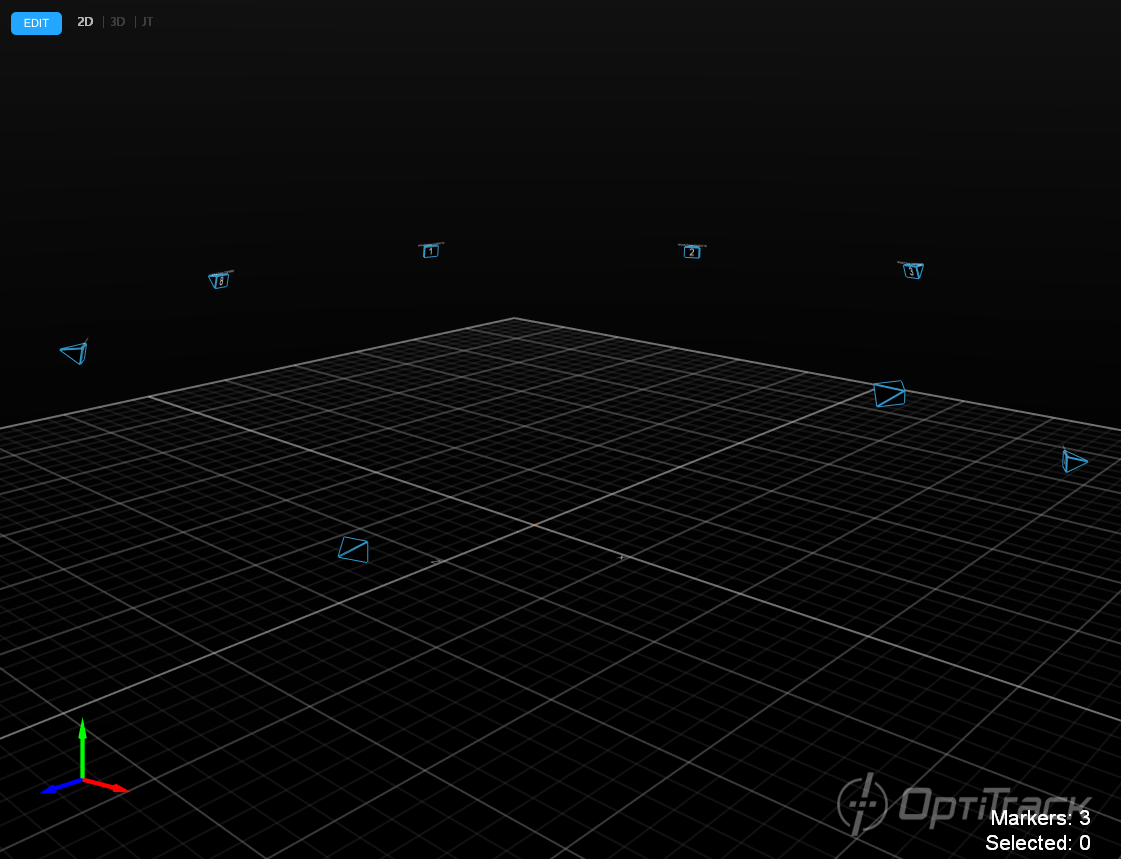

Supplement: Supplemental Information 3 — The 8 camera montage (from the side) as seen from Optitrack’s Motive software. [file peerj-06-6003-s003.png]

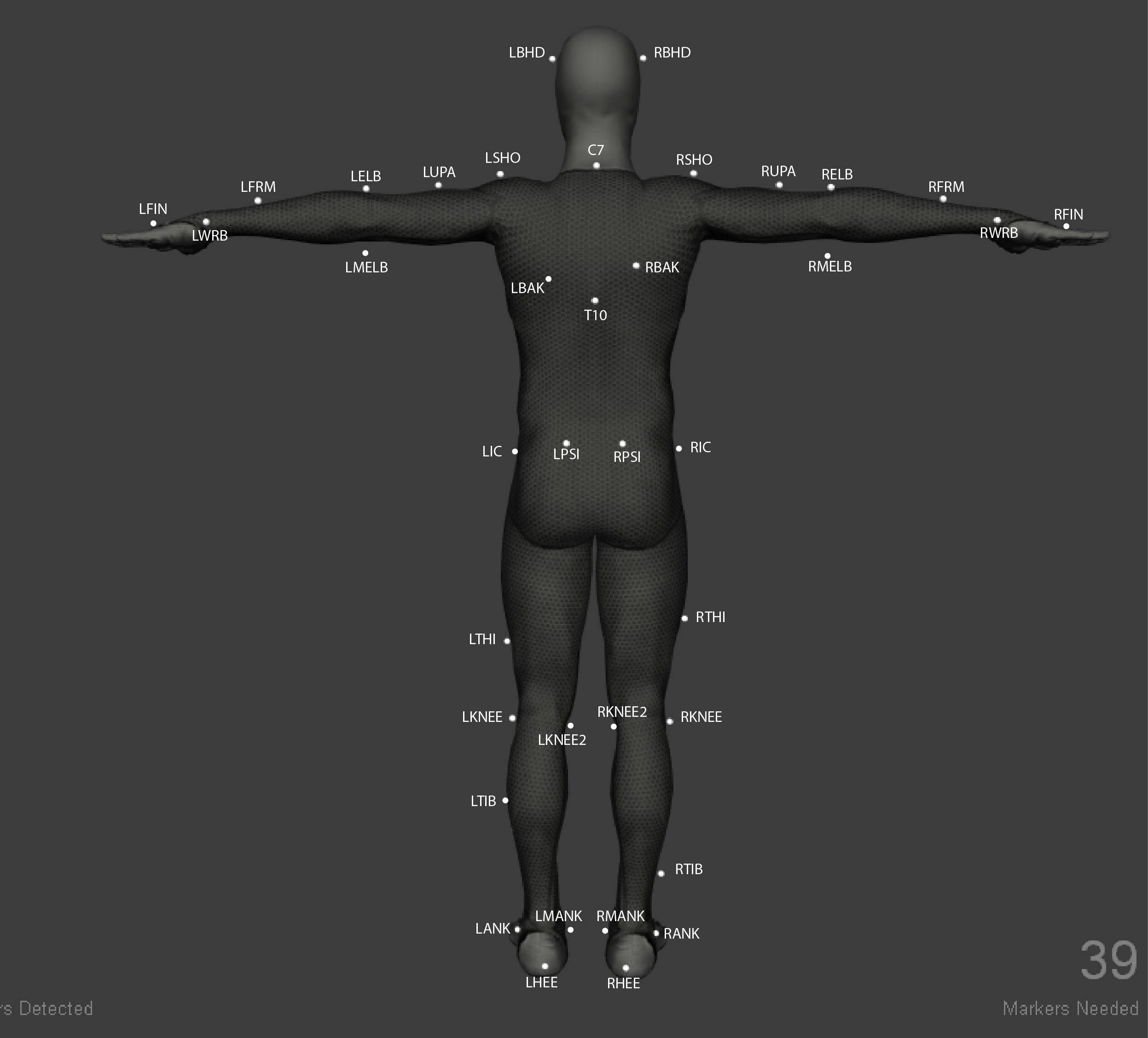

Supplement: Supplemental Information 4 — The front anatomical view of how body markers were placed on subjects. [file peerj-06-6003-s004.png]

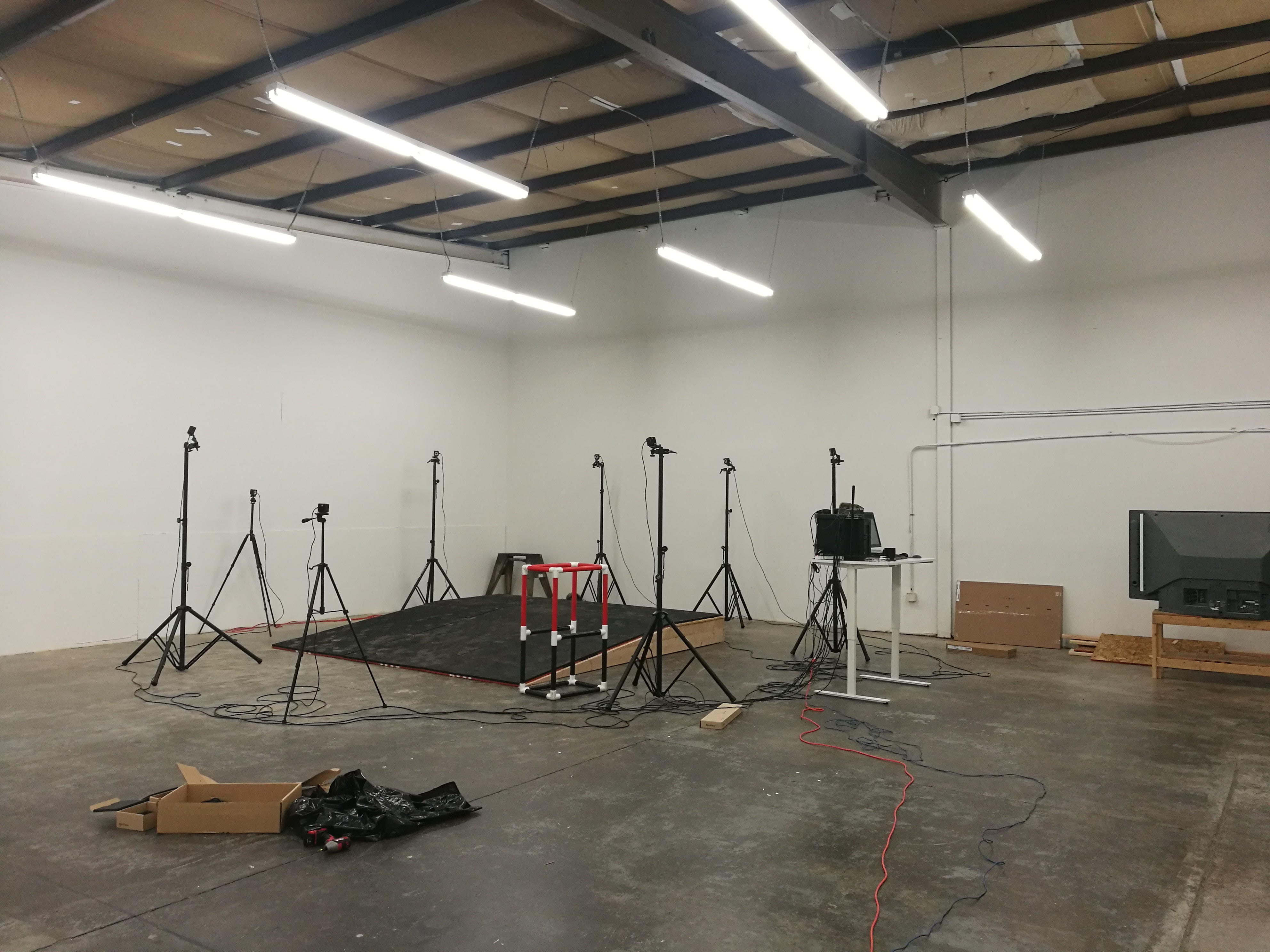

Supplement: Supplemental Information 5 — Physical view of the 8 Optitrack camera montage. [file peerj-06-6003-s005.jpg]
