# Supplementary material for: Effects of a six-week weighted-implement throwing program on baseball pitching velocity, kinematics, arm stress, and arm range of motion
Source: PeerJ. 2018 Nov 23;6:e6003. doi: 10.7717/peerj.6003 (PMC6254244; doi:10.7717/peerj.6003)
Supplement: Supplemental Information 6 — Referenced as HTKC1 in the study, these are exercise guidelines. [file peerj-06-6003-s006.pdf]

Foam Rolling

Utilizing the foam roller is generally the first thing our athletes will use for SMR because it's less abrasive than other trigger-specific tools, making it an ideal way to start off soft-tissue work. In chronological order, the following cover a few primary areas our athletes use the foam roller on:

1. Calves/Lower Legs

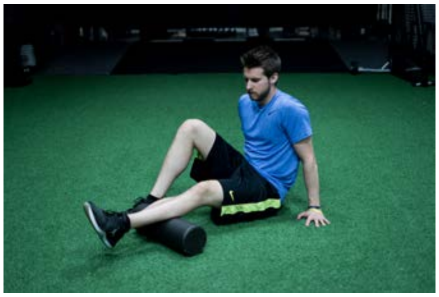

OVERVIEW

The calves and the muscles of the lower legs are responsible for movement at the ankle and knee. There are multiple groups of muscles, but they can all be hit in a few passes of a foam roller with creative positioning of the lower leg. By rolling out the lower legs, good flexibility and mobility of the ankle and knee is retained, optimizing the stride and blocking phases of the pitching delivery.

METHOD

Have an athlete sit on the ground and place the foam roller underneath the calf muscle. Once everything is in position, place the hands on the ground and lift the body slightly off the ground to create a little bit of pressure on the calf muscle. Once the pressure feels right, the athlete should maneuver the body in a manner that allows the roller to cover the desired surface area of the calf muscle. The athlete can slightly internally and externally rotate the leg to cover different parts of the calf muscle. Once the calf muscle on one leg has been rolled out for an appropriate amount of time, the athlete should repeat the same steps on the other leg. If the athlete has more concentrated spots of soreness or tightness, then it may be beneficial to use a LAX ball or the Stick to roll it out during the LAX ball rolling phase of the warm-up.

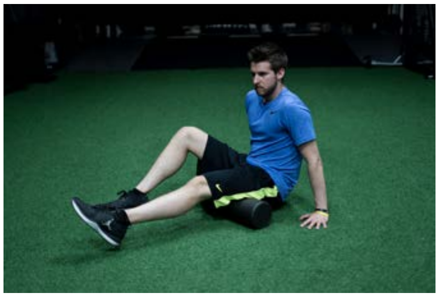

2. Hamstrings

OVERVIEW

The hamstrings are a group of muscles and tendons that are primarily responsible for flexing the knee and extending the hip. Their secondary function involves medially rotating the hip inwards. By keeping this muscle group pliable and free of adhesions, it ensures consistent stride direction, stride power, lead-leg blocking strength, and lower-half rotation.

METHOD

An effective method to roll out the hamstrings is almost identical to the technique for rolling out the calves/lower legs. The main difference is the location of the foam roller, which should be placed underneath the hamstring. If an athlete has more concentrated spots of soreness or tightness, the Stick is likely a better option than a LAX ball to work on that spot. To roll out hamstrings with the Stick, have the athlete sit down with the knees slightly bent to create space between the hamstrings and the ground, but also to minimize hamstring contraction. Once correctly positioned, take the Stick and roll out the hamstrings, adding and subtracting pressure and covering as much surface area as needed.

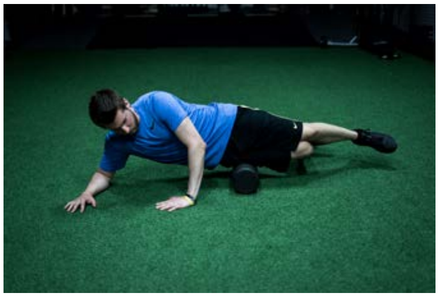

3. IT Band + Tensor Fascia Latae (TFL)

OVERVIEW

The iliotibial (IT) band supports movement around the knee. It often gets tight in athletes who move explosively laterally and therefore is a prime target for SMR. The tensor fascia latae (TFL) and surrounding musculature aids hip flexion, hip adduction, and hip internal rotation. The TFL is connected to the IT band. By keeping the IT band free of adhesions, knee pain is far less likely to occur. Quality of the blocking phase will increase if the IT band works properly. By keeping the TFL freed up, lateral explosiveness can be retained without risking strains in the lower half. If lateral-knee pain is present, rolling out the TFL and IT bands should be the main targets.

METHOD

First, place the foam roller on the ground, then lie down sideways, specifically placing the middle of the IT band on top of the foam roller. Once appropriately positioned, the athlete should place the hands on the ground for both increased stability and ability to move. Once stabilized, the athlete should use the arms to propel the body back and forth enough to allow the foam roller to cover a comfortable amount of surface area. After this is done, flip over to the other side and repeat the same steps. If the athlete notices more concentrated spots of soreness, light trigger-point work during the LAX ball-rolling phase of the warm-up may be helpful. To avoid putting too much pressure on the IT band with the LAX ball, the best technique is for the athlete to sit on the ground and roll the LAX ball over the area of soreness with a hand, which provides better accuracy and more ability to control the amount of pressure in the area.

4. Quadriceps

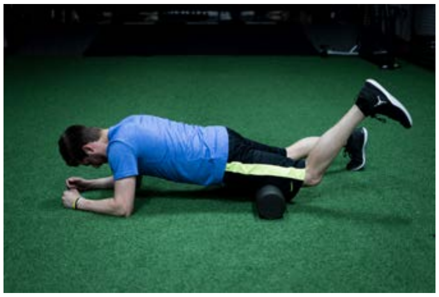

OVERVIEW

The quadriceps are responsible for knee extension and hip flexion. By rolling out the quads on a regular basis, hip flexion can be optimized during the pitching delivery, allowing increased opportunity for good trunk flexion and forward shoulder rotation.

METHOD

The technique used to effectively roll out the quadriceps is almost identical to the technique used to roll out the IT bands, with the main exceptions being where the foam roller is placed and the exact positioning of the body. Instead of lying down sideways, the athlete should lie down facing the floor with the foam roller placed in between the ground and the middle of one of the quadriceps. The arms should then be utilized to move the athlete back and forth, in the same manner as rolling out the IT bands. If more concentrated spots of soreness still exist once the foam roller has covered both quads, it's recommended for the athlete to sit down and with the legs flat on the floor in a relaxed position and use the Stick to roll out the particular spot. Once correctly positioned, take the Stick and roll out the quads, adding and subtracting pressure and covering as much surface area as needed.

5. Glutes

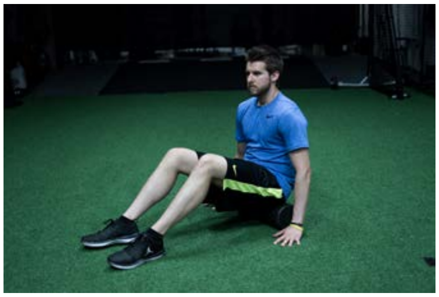

OVERVIEW

The glutes dominate total hip movement, including extension, abduction, and external rotation. By rolling out the glutes, a neutral and slightly extended lumbar posture is easier to maintain throughout the pitching delivery, reducing the chances of lower-back pain. Additionally, force is better transmitted through hip extension and rotation if the glutes are firing properly.

METHOD

First, have the athlete sit on top of the foam roller, with the feet flat on the floor, legs bent, and hands on the floor to stabilize the upper body. After the athlete has reached a good starting position, the next step is to use the legs to move the foam roller back and forth over the surface area of the glutes. The glutes do not have the same amount of length as the parts of the legs discussed before, so the back and forth movement should be very short. To laterally cover the entire surface area of the glutes, the athlete can use the core to slightly rotate the hips to cover the outside portions of the glutes. If the athlete detects concentrated areas of soreness or discomfort once foam rolling is complete, using a LAX ball to further roll out these areas is recommended during the LAX ball-rolling phase of the warm-up. Gluteus medius tightness and pain in particular is not uncommon to find in pitchers, so sometimes spending an extra 30-45 seconds on that area can be greatly beneficial.

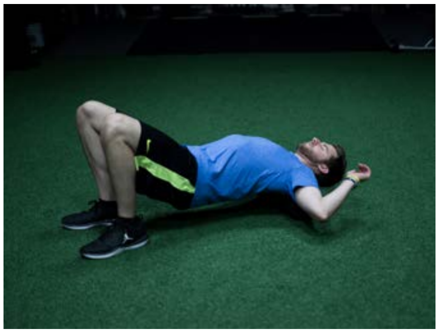

6. Thoracic Spine (Upper Back)

OVERVIEW

The thoracic spine is the middle/upper vertebrae in the spine. By mobilizing the thoracic spine, the torso is free to rotate quickly, which is one of the most significant components of ball velocity.

METHOD

With the foam roller placed on the ground, have the athlete sit on the ground so that when he lays his back down, the shoulder blades touch the foam roller. Make sure the legs are slightly bent and feet are flat on the floor as well. After this position has been reached, the athlete should use the glutes and abs to lift the butt off of the floor, just enough to place pressure against the thoracic spine, but not enough to arch the back. Once the correct starting position is established, use the legs to propel the foam roller back and forth over the thoracic spine, covering the specific surface area between the mid-back to the base of the neck. *Do not* roll the lower back (lumbar spine).

LAX Ball Rolling

Once foam rolling is done on the larger muscle groups, our athletes go through a secondary set of rolling, this time with a LAX ball to target smaller muscle groups and more concentrated areas of soreness and/or tightness. In chronological order, the following cover a few primary areas we suggest using LAX balls on:

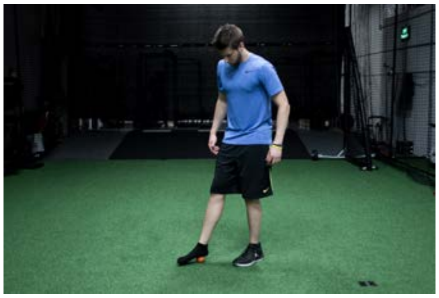

1. Plantar Fascia

OVERVIEW

Plantar fascia connects the toes to the Achilles tendon. Rolling out the bottom of the foot helps maintain a healthy amount of ankle mobility. Since the plantar fascia starts all movement that requires extension of the lower half, it's a critical area to keep pliable.

METHOD

With the LAX ball on the ground, make sure the athlete's shoes are off before placing the middle of the foot on the ball. Once a comfortable amount of pressure is applied, the athlete should move the foot around in a manner that the ball covers the full surface area of the bottom of the foot. Once completed, repeat the same steps on the opposite foot.

2. Medial Scapula Crest

OVERVIEW

The posterior shoulder stabilizes the shoulder joint and supports internal rotation. Mobilizing the medial scapula crest will help the rotator cuff function at a higher level, improving opportunity to produce better velocity and reducing the chance of soft-tissue injury around the shoulder.

METHOD

The two primary ways to roll out the medial scapula crest are lying on the ground and standing up against a wall. The latter option is a good way for athletes to become acquainted with using trigger-specific tools like a LAX ball, since lying on the ground creates more pressure on the targeted areas. Whether the athlete is standing up or lying down, the basic technique for rolling out the medial scapula crest is the same. Have the athlete pin a LAX ball between the scapula and spine on the throwing side and have the athlete roll the ball up and down a couple inches in each direction. To add some variance, come to a stopped position and stabilize the LAX ball in various spots, and reach overhead and across the body to mobilize the shoulder.

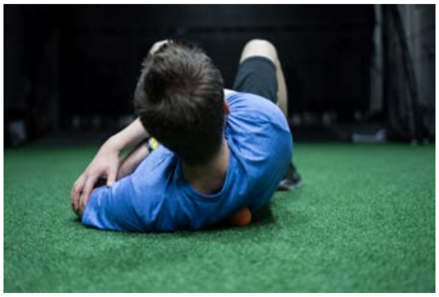

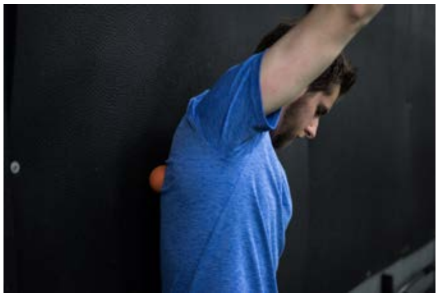

3. Teres Minor/Infraspinatus

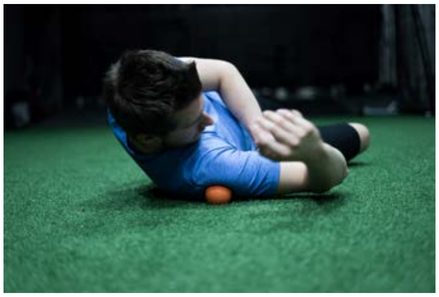

OVERVIEW

The teres minor and the infraspinatus are two muscles of the rotator cuff that actively externally rotate the shoulder. These muscles also support deceleration of the throwing arm during the pitching delivery. By mobilizing this group, keeping the forearm laid back in MER during the Driveline Phase will become easier and deceleration will be optimized during the Recovery Phase.

METHOD

The recommended position for rolling out the teres minor/infraspinatus is for athletes to lie on their back and place the LAX ball between the bottom of their throwing-side shoulder blade and the floor. Controlling pressure application should be much easier when rolling out these muscles than the medial scapula crest, so there shouldn't be a need to stand up. Slowly roll the ball in a small circular motion by moving the torso around, stopping occasionally on sore spots and taking the throwing arm through external and internal rotation while the ball is pinned.

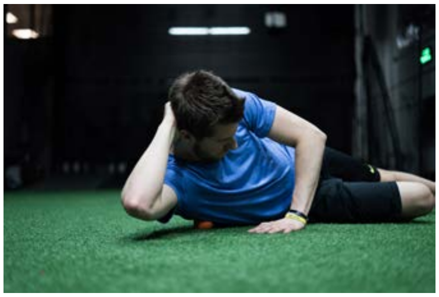

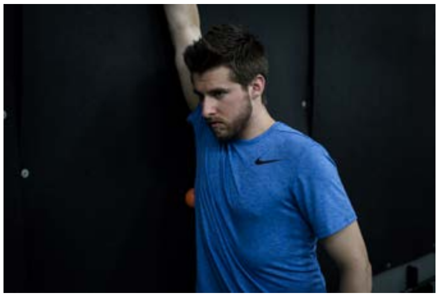

4. Latissimus Dorsi (Lats)

OVERVIEW

The lats have a huge cross-section and support deceleration of the throwing arm, as well as internal rotation, extension, and adduction. Rolling out the lats makes it easier for athletes to maintain a fairly low elbow position towards the end of the Pickup Phase and at the beginning of the Elbow-Spiral Phase. Pitchers that have trouble with the elbow climbing too high generally tend to have tight lats, coupled with mechanical inefficiencies.

METHOD

Athletes will most effectively roll out their lats by standing up, with their throwing-side lat against a wall, and placing the LAX ball between the middle of the throwing side lat and the wall. After raising the throwing arm overhead, they should next use their legs to move the ball up and down the side of the throwing side lat. The athlete may also roll out the opposite lat if desired, though this is not required.

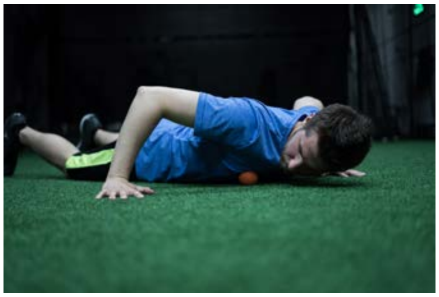

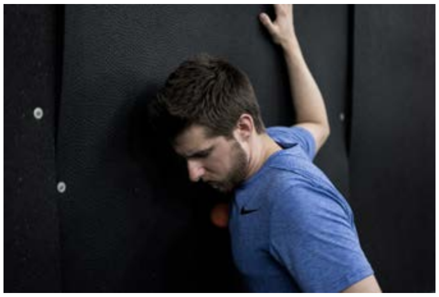

5. Pecs/Chest

OVERVIEW

The pectoralis major/minor are responsible for horizontal adduction and internal rotation of the throwing shoulder and often get tight in all baseball pitchers, regardless of mechanical efficiency. Rolling out shortness in the pecs can improve posture and create a more significant delay in internal rotation during the Driveline Phase of the pitching delivery. Anterior shoulder stress will be reduced if the pecs are properly supporting the shoulder-scapular complex.

METHOD

The most pressure will be applied to the pecs while lying down, but admittedly it is an awkward position and not all athletes will feel like they get optimal value out of lying down while rolling out their pecs. Because of this, we also recommend the options of standing up against a wall, or even using the opposite hand to roll the LAX ball against the throwing-side pec muscle. If not utilizing any hands to roll the LAX ball, have the athlete pin the LAX ball between the middle of the throwing-side pec and the surface chosen to use (ground or wall). After that, the athlete should maneuver the body so that the ball rolls against the pec in a circular motion. If the athlete chooses to roll out the throwing-side pec with the opposite hand, the athlete should simply sit down and pin the LAX ball to the middle of the pec with the opposite hand. Once the starting position is obtained, roll the ball around in a circular motion, adding or subtracting pressure as needed to free up any concentrated areas of soreness and/or tightness.

---

6. Triceps

OVERVIEW

The triceps extend the elbow, one of the most critical movements in the pitching delivery. Rolling out the triceps on a regular basis can maintain range of motion at the elbow, which promotes a healthy arm and proper transfer of force midway through internal rotation in the last moments before the ball exits the hand.

METHOD

While standing up against a wall to roll out the triceps can work, we recommend lying down for this one. With the ball placed on the ground, the athlete should lie down on the stomach and, with a bent elbow, place the middle of the tricep on top of the LAX ball. From there, the athlete should maneuver the body to roll the ball up and down the tricep, adding or subtracting pressure as needed to free up any concentrated areas of soreness and/or tightness.

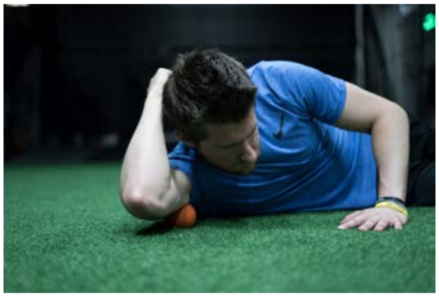

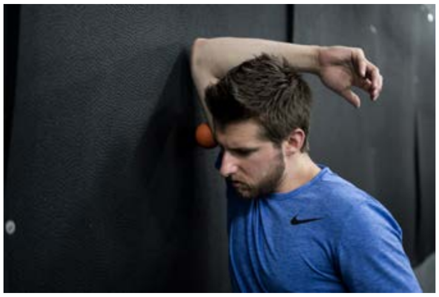

7. Forearms

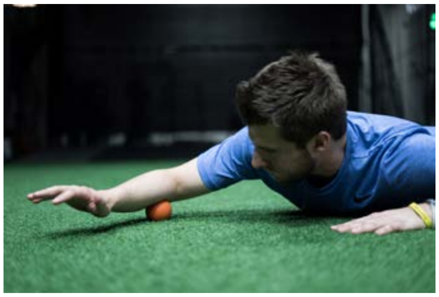

OVERVIEW

The forearm flexors and extensors help protect the elbow during the pitching delivery, absorbing a significant amount of force the ulnar collateral ligament (UCL) would otherwise take on. Mobilizing this group can make the transition between supination and pronation significantly easier during internal rotation. By restoring a pliable surface in the flexor-pronator bundle, stress can be reduced on the UCL and the posterior bony structures of the elbow.

METHOD

Athletes can adequately roll out the throwing-side forearm with a LAX ball by either lying down on the stomach or standing against a wall; the technique for both is essentially the same. In both cases, athletes can vary how much they lean their bodyweight onto their forearm to add or subtract pressure. After a fairly high, but tolerable amount of pressure is obtained, simply move the arm in a back and forth motion to roll the LAX ball up and down the forearm. If more work is necessary, one additional idea is to pin the Stick against a power cage/squat rack and roll the forearm up and down the surface of the Stick while pronating and supinating at the forearm to capture the entire area.

2

RESISTANCE BANDS:
Shoulder and Elbow Care

With loads of a contradictory baseball information circling the internet in 2017, it's nearly impossible to get pitchers to agree on anything regarding pitching training. However, there's one training modality that all sides mutually agree upon: resistance band (aka surgical tubing) exercises. Found in bullpens and training rooms around the world, these bands can be used to great effect.

Choosing the correct exercises is important—every movement should drill a specific purpose. For warming up, activating the external rotators and biceps is critical; while for cooling down, getting blood flow to the shoulder in general to jumpstart the recovery process is a primary factor. Simply pulling the bands in random directions won't help.

These exercises should be done in a controlled fashion with a one-second cadence and no pause. That means the concentric position of the movement should take one second and the eccentric portion should take one second. For example, an internal-rotation exercise is one internal rotation (hand moves down) followed immediately by a one-second external rotation (hand moves up).

Your resistance bands should be durable and easy to carry around. We highly recommend the **Jaeger Sports J-Bands**, as they come with wrist cuffs to allow less restricting movement by preventing the need to grasp at handles. Additionally, the carabiner allows for easy hookup to any fence without needing to loop the band into the fence, thus extending the life of the resistance bands.

1. Forward Fly to Overhead Reach

BENEFIT

Activation of the anterior shoulder prior to throwing helps to ensure proper function of the rotator cuff during throwing. Getting the arms overhead warms up the shoulder-scapular complex in one-compound movement.

METHOD

With the cuffs secured around the wrists, have the athlete stand facing away from the anchor point of the resistance bands. Walking forwards or backwards can increase and decrease resistance. Once a comfortable starting point resistance is established, make sure the feet are roughly shoulder width apart, with a slight bend at the knees and the hips, and the abs are braced. With the thumbs pointing up and arms straight out to the side at shoulder height, have the athlete bring the arms forward and to the front (forward fly). Do not bring the hands together; once the arms are straight in front of the shoulders, the athlete should raise the hands straight up (overhead reach), keeping the palms of the hands facing each other. When raising the hands upward, make sure the elbows stay straight. Elbow flexion is not a substitute for strength. Once the end range of motion is reached, have the athlete lower the hands back down to shoulder height then control the arms back to the starting position. Repeat these steps for the prescribed number of reps.

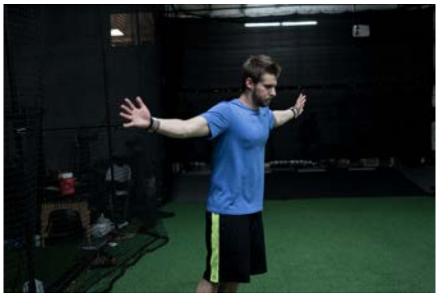

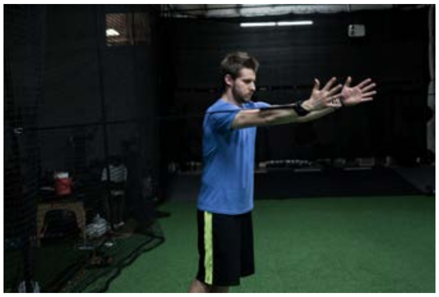

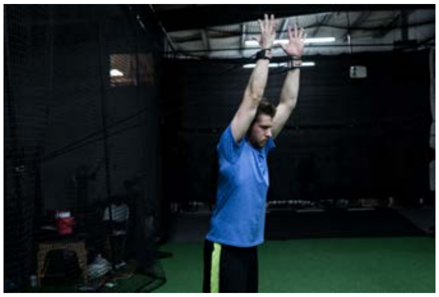

This chapter discusses the benefits and methods behind each exercise we perform in our J-Band series for warm-up and recovery work. Each of the following exercises are listed out in the typical chronological order that our athletes perform them.

WARNING:
Do not clip J-Bands at face height for any exercise!

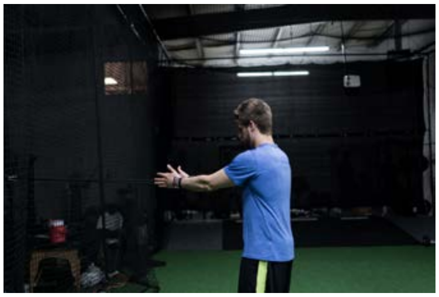

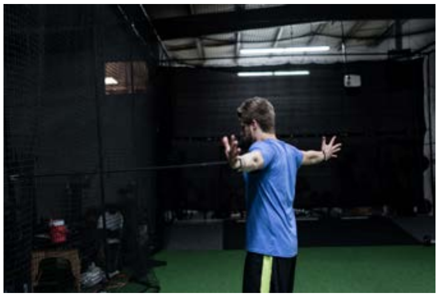

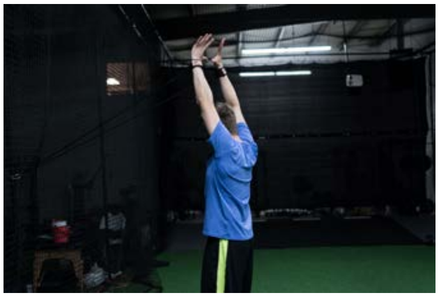

2. Reverse Fly to Overhead Reach

BENEFIT

Activation of the posterior shoulder prior to throwing helps to ensure proper function of the rotator cuff during throwing. Getting the arms overhead warms up the shoulder-scapular complex in one-compound movement.

METHOD

With the cuffs secured around the wrists, have the athlete stand facing the anchor point of the resistance bands. Walking forwards or backwards can increase and decrease resistance. Once a comfortable starting point resistance is established, make sure the feet are roughly shoulder width apart, with a slight bend at the knees and the hips, and the abs are braced. With the thumbs pointing up, and the arms straightened in front of the athlete at shoulder height, have the athlete bring the arms backwards until they are directly to the side of the body (reverse fly). Once the arms are straight out to the side, the athlete should then raise the arms overhead with the palms facing forward, eventually touching the thumbs together at the top, if possible (overhead reach). Make sure to keep the arms straight during the overhead reach. Once the overhead end range of motion is reached, have the athlete lower the arms back down to the side at shoulder height and then control the arms back to the starting position. Repeat these steps for the prescribed number of reps.

3. Bicep Curls with Supination

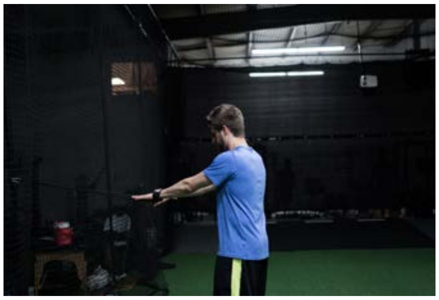

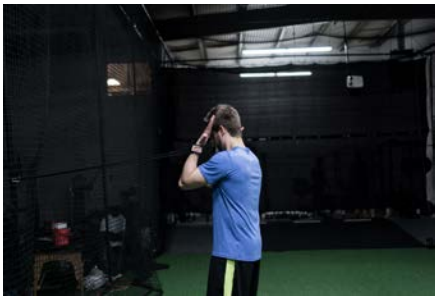

BENEFIT

Concentric work of the biceps prepares the muscles for deceleration in the pitching delivery. This movement also helps deliver increased blood flow to the area, making it an excellent recovery movement.

METHOD

With the cuffs secured around the wrists, have the athlete stand facing the anchor point of the resistance bands. Once the appropriate distance away from the anchor point is established, the athlete should start with the arms straight in front and pronated (palms down). The next step is to supinate the forearms (turn the palms up) while curling the bands towards the face. Once the end range of motion at the top is reached, unwind the arms back to the starting pronated position in a controlled manner. Repeat these steps for the prescribed number of reps.

4. Tricep Extension with Pronation

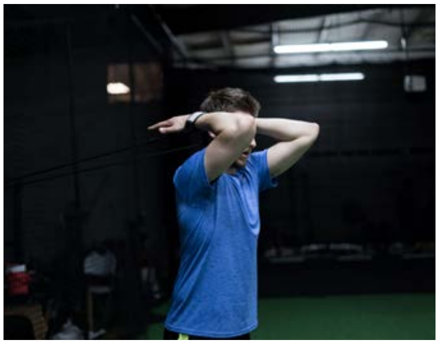

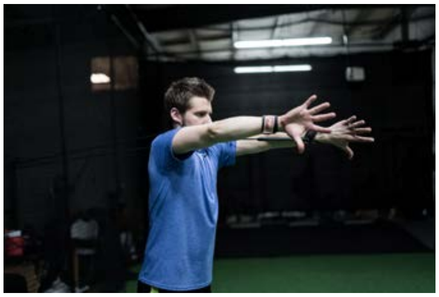

BENEFIT

Concentric work of the triceps prepares the muscles for acceleration in the pitching delivery. This movement also helps deliver increased blood flow to the area, making it an excellent recovery movement.

METHOD

With the cuffs secured around the wrists, have the athlete stand facing away from the anchor point of the resistance bands. Once the appropriate distance away from the anchor point is established, the athlete should start with the elbows up and the hands to the side of the ear, palms facing the shoulders (thumbs away from the head). The next step is to extend the arms to the front at head height, pronating the forearms in the process (turning the thumbs toward the ground, palms out). Once the end range of motion at the front is reached, reverse the original movement in a controlled manner back to the starting position. Repeat these steps for the prescribed number of reps.

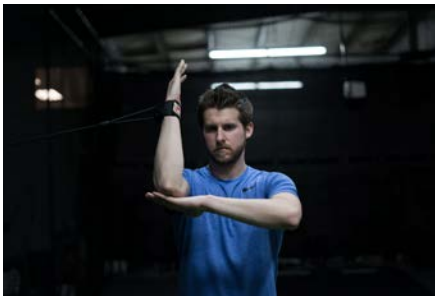

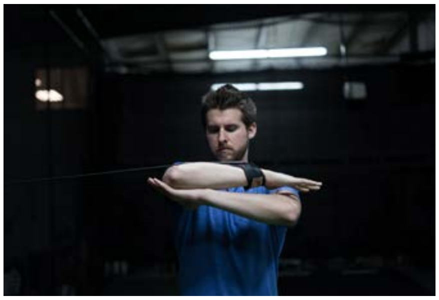

5. Internal Rotations

BENEFIT
Concentric work of internal rotators prepares the muscles for arm acceleration in the pitching delivery. This movement also helps deliver increased blood flow to the area, making it an excellent recovery movement.

METHOD
With a cuff secured around the wrist on the throwing arm, the athlete should stand sideways, with the throwing arm facing the anchor point of the resistance bands. Raise the elbow to the front at shoulder height with the forearm in a vertical position. Place the back of the opposite hand underneath the throwing elbow to keep the arm stable, creating the ability to properly isolate the internal rotation movement. Once the starting position is fully established, take the palm of the throwing arm down to the opposite elbow by rotating at the shoulder (internally rotate). Once internal-rotation end range of motion is reached, control the forearm back to the original vertical position. Repeat these steps for the prescribed number of reps.

6. External Rotations

BENEFIT
Concentric work of external rotators prepares the muscles for arm acceleration in the pitching delivery. This movement also helps deliver increased blood flow to the area, making it an excellent recovery movement.

METHOD
With a cuff secured around the wrist on the throwing arm, the athlete should stand sideways, with the glove side arm facing the anchor point of the resistance bands. Raise the elbow to the front at shoulder height with the forearm in an internally rotated horizontal position. Place the back of the opposite hand underneath the throwing elbow to keep the arm stable, creating the ability to properly isolate the external rotation movement. Once the starting position is fully established, have the athlete lift the forearm to a vertical position by rotating at the shoulder (externally rotate), without letting the humerus drift off to the side of the athlete. Once external-rotation end range of motion is reached, rotate the forearm back down to the starting position in a controlled manner. Repeat these steps for the prescribed number of reps.

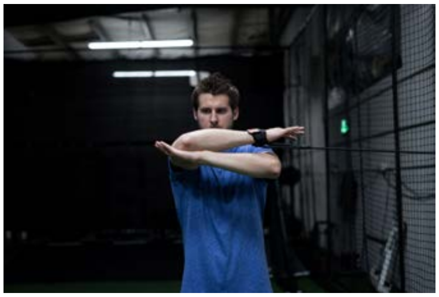

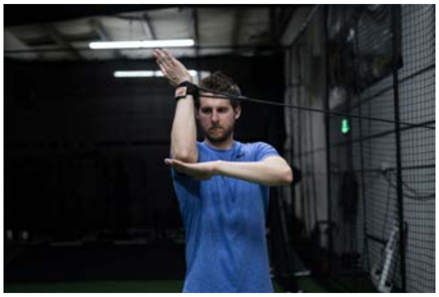

3

OSCILLATION THERAPY:
Dynamic Stabilization

Rhythmic stabilization and oscillation therapy have long been staples of the physical-therapy world for rehabilitation purposes. By training the contractile tissues of the shoulder-scapula complex, directly and indirectly working the joint and surrounding soft tissues, the entire area must respond to rapid positional changes.

A few tools used for the individual oscillation-therapy exercises in *Hacking the Kinetic Chain — Advanced Pitching* are

- *Oates' Specialties Shoulder Tube*
- *Fitryo Total Bar*

A popular product on the market is the Bodyblade, but due to its flat shape, the Bodyblade does not work well in multiple directions and therefore is overly restrictive. If you have one, it can be used for the exercises in this chapter, but efficiency will be significantly lower.

By increasing the blood flow to the shoulder-scapula complex, the body becomes more prepared for physical activity, making it an excellent warm-up tool. Stimulating proprioceptors through use of oscillation therapy provides great benefits if used prior to an intense workout—it is also a helpful addition to a post-throwing recovery protocol. If athletes do not have their own oscillation therapy routine, this chapter outlines the exercises our athletes use, as well as the chronological order in which they are performed.

1. Shoulder Flexion in Front

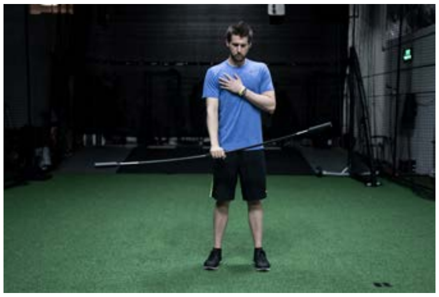

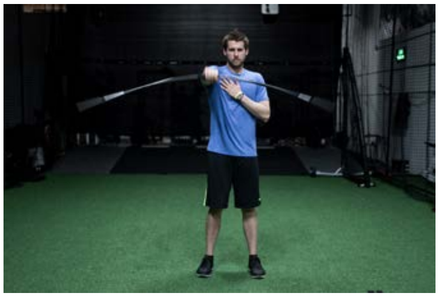

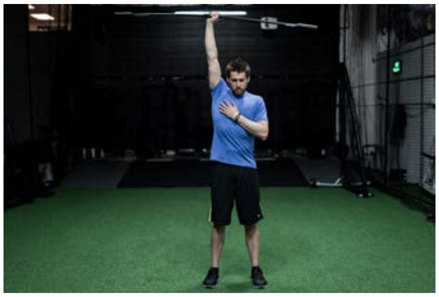

METHOD

Shoulder Flexion in Front ("Front Raises") is done with the athlete standing square with feet shoulder-width apart and the throwing hand holding the implement with the palm down in front. The athlete should start bouncing the implement up and down in front of the body by shaking the throwing arm towards the sky and the ground in rhythmic strokes. While still bouncing the implement up and down, the arm should be raised overhead until the end range of motion is reached. Once the end range of motion has been reached at the top, the athlete should reverse course and slowly bring the hand back down to the starting position. It should take roughly 4-5 seconds to take the hand in each direction. Repeat these steps for the prescribed number of reps.

2. Shoulder Abduction to the Side

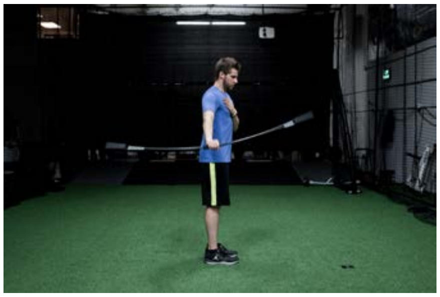

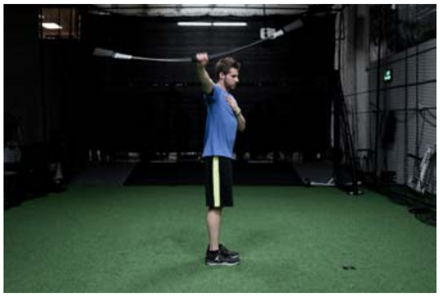

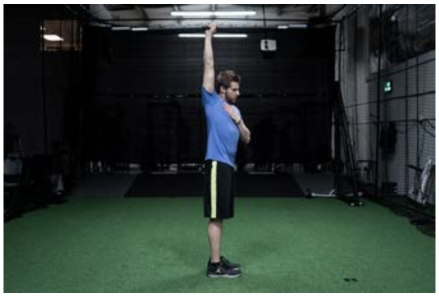

METHOD

Shoulder Abduction to the Side ("Side Raises") is done with the athlete standing square with feet shoulder-width apart and the throwing hand holding the implement with the palm down to the side. The athlete should start bouncing the implement up and down to the side of the body by shaking the throwing arm towards the sky and the ground in rhythmic strokes. While still bouncing the implement up and down, the arm should be raised overhead until the end range of motion is reached. Once the end range of motion has been reached at the top, the athlete should reverse course and slowly bring the hand back down to the starting position. It should take roughly 4-5 seconds to take the hand in each direction. Repeat these steps for the prescribed number of reps.

3. External/Internal Rotations in Close

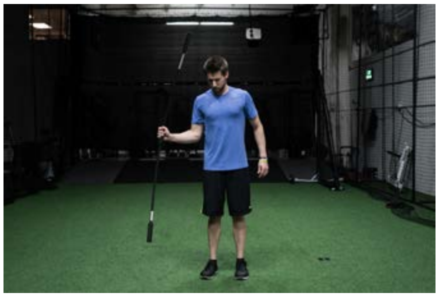

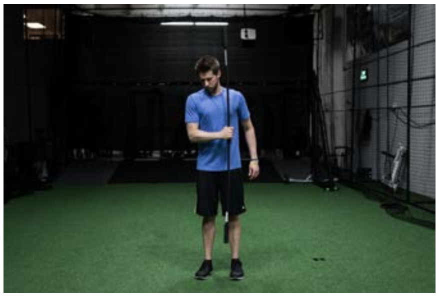

METHOD

External/Internal Rotations in Close ("Side to Sides") are done with the athlete standing square with the feet shoulder-width apart and the throwing hand holding the implement with the thumb up (supinated) in front and with the elbow close to the rib cage. The athlete should start bouncing the implement from side to side by shaking the throwing arm toward the left and right in rhythmic strokes. While still bouncing the implement, the arm should slowly internally rotate until the end range of motion is reached. Once the internal-rotation end range of motion is reached, the arm should slowly externally rotate until the end range of motion is reached on that side. It should take roughly 4-5 seconds to take the hand in each direction. Repeat these steps for the prescribed number of reps.

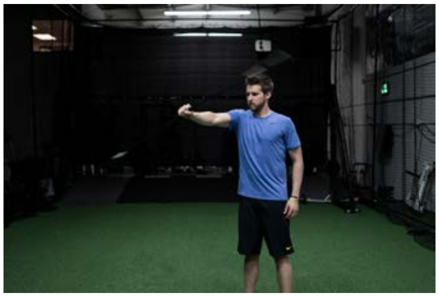

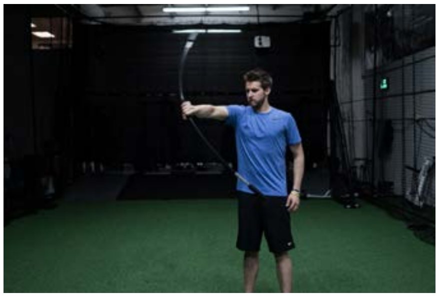

4. Pronation/Supination Twirls

METHOD

Pronation/Supination Twirls are done with the athlete standing square with the feet shoulder-width apart while holding implement with palm facing up and the throwing arm straight out in front of the body. While the elbow will flex a little bit, the athlete should make the effort to shake the implement in and out by moving the arm in the same manner with the shoulder. Once this movement has been established, the next step is for the athlete to slowly pronate the forearm (turn the thumb down) while still shaking the implement. Once the end range of motion is reached for pronation, slowly reverse course to return the hand to the starting supinated position. It should take roughly 4-5 seconds to turn the hand in each direction. Repeat these steps for the prescribed number of reps.

5. Stride-Length Forward Shoulder Rotations

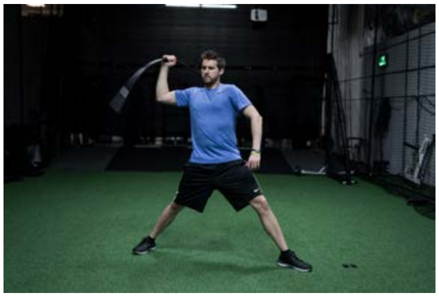

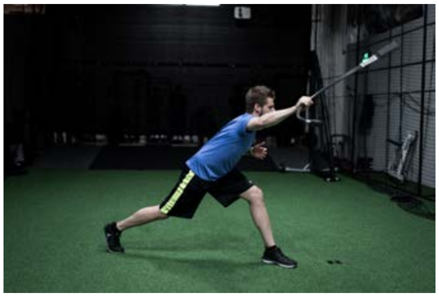

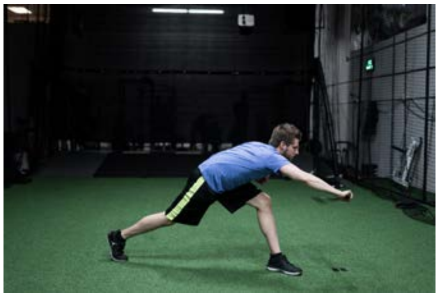

METHOD

Stride-Length Forward Shoulder Rotations are done with athlete starting in the same stance as used for Rocker Throws (see Rocker Throws in the PlyoCare drills chapter for stance explanation). Once the stance is established, the athlete should place the throwing-arm elbow slightly below the shoulder, with the forearm in a vertical position and the hand in a slightly supinated position. It's worth noting that in a perfect world we would prefer the athlete to start in a neutral position since it more accurately represents good hand position at stride-foot contact, but in this case, starting with a neutral hand would cause the athlete to hit himself in the head with the implement. A slightly supinated hand position helps solve this problem for the purpose of this exercise. After the starting position is fully established, the athlete should start bouncing the implement up and down by moving the arm in the same manner. Once the implement movement has started, rotate the throwing shoulder towards the lead foot while still bouncing the implement. Once the trunk flexion end range of motion has been reached, slowly counter-rotate back to the starting position while continuing to bounce the implement. It should take about 10-15 seconds for the full movement to occur. Repeat these steps for the prescribed number of reps.

4

WRIST WEIGHTS:
Overload Corrections

Light dumbbells have been used in baseball for rehabilitation and catch-all "shoulder programs" for decades. While these modalities have their place, wrist weights are far more effective from a dual-factor perspective. Wrist weights work well at generating a physiological response and also at teaching the body to move more efficiently through an overloaded range of motion.

Wrist weights are "thrown" in a ballistic fashion while being loosely held in the hands. The ideal grip is to have the fingertips slightly over the wrist weight for support while maintaining a relaxed forearm throughout the exercise. Wrist-weight drills are used to train optimal unwinding of the arm from the Driveline Phase, teaching how to efficiently move from external rotation with a slightly supinated forearm to internal rotation with a pronated forearm.

However, attempting to actively contract the pronator-flexor mass to aid this process will only cause timing problems due to the incredibly quick speed at which arm acceleration occurs. Using overloaded implements like wrist weights can build efficient technique into the mechanical map of the neurological system, ensuring that it will be integrated into the delivery when combined with other drills provided in *Hacking the Kinetic Chain — Advanced Pitching*.

Continuing to hold on to the wrist weights throughout the ballistic motions helps decrease deceleration stress since there is no separation of the

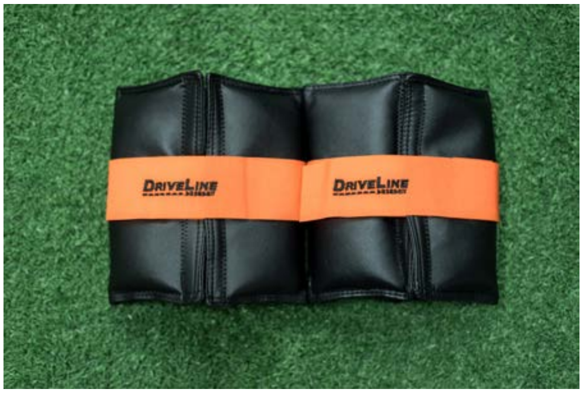

weighted implement from the hand. Instead of a completely reactionary deceleration phase, the ability to feel the mechanical pattern provides very strong proprioceptive feedback, which is perfect for attempting to retrain movement patterns.

While the 5-kg wrist weight is carried in external rotation and forearm layback, the shoulder experiences a similar inertial mass when a baseball is thrown. Consider that a 5-oz. baseball is accelerated in external rotation as a result of the torso's rapid angular velocity, literally "gaining weight" as the forearm is pushed backwards in the loaded position during the Elbow-Spiral Phase. Simulating this end range of motion while maintaining a loose forearm allows the athlete to feel how arm acceleration actually occurs from the "loaded" position, making wrist weights an invaluable training tool for pitchers.

One common mistake trainees make is undervaluing the use of a wrist weight on the glove hand. An overloaded glove arm gives the athlete more proprioceptive feedback for how the non-throwing arm creates positive disconnection to leverage trunk rotation, making the use of a wrist weight on the glove hand very instructional. The quicker and more efficiently the glove arm moves around the torso while the throwing arm begins to load, the more kinetic energy becomes available while straightening out the line of force application. Think of the ideal path of the throwing-arm wrist as looking something like an oval around the torso when viewed overhead, rather than a circle.

The further away the focal points of the ellipse are from one another, the straighter *and* longer the Driveline Phase becomes, reducing stress on the elbow and increasing total force applied to the baseball. Also, consider that when a baseball is released, the trajectory can be drawn like a line tangent to the arc—the single intersection of the two lines representing the "release point" of the ball.

Wrist weights are currently the single best modality to train this path while providing unique physiological and kinesthetic stimuli. This chapter outlines four wrist-weight exercises our athletes use, and the chronological order in which they are performed.

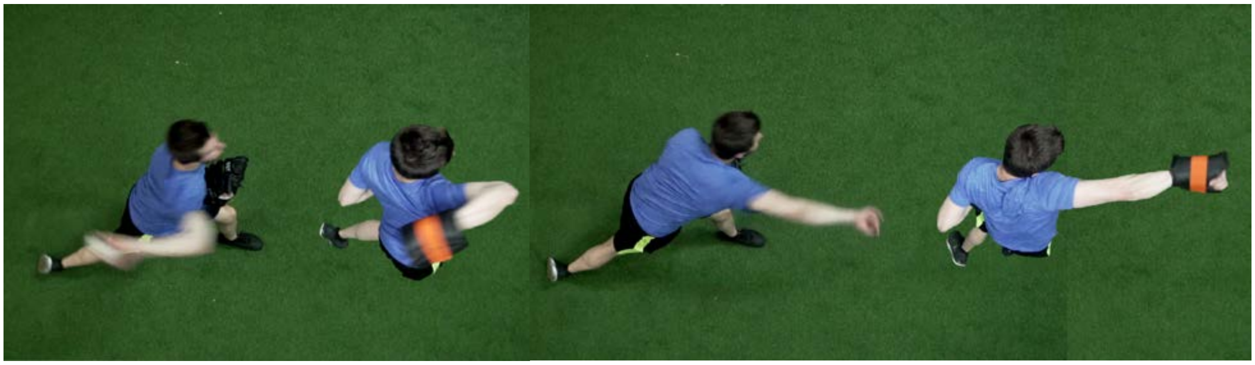

1. Pronated Swings

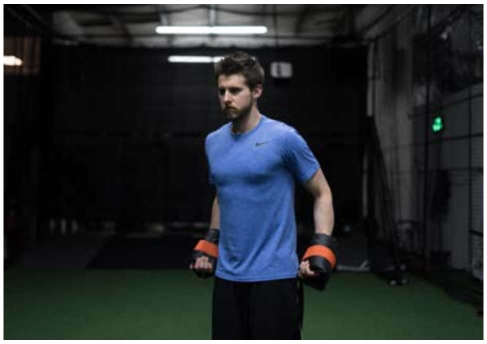

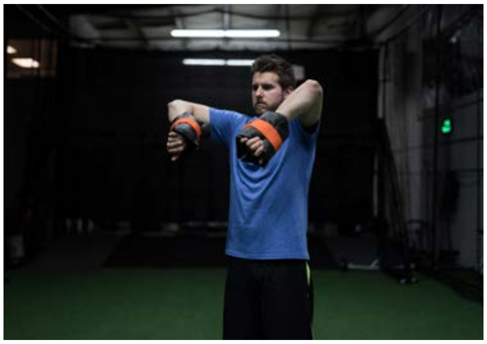

OVERVIEW

The least complex wrist-weight exercise out of the bunch, Pronated Swings are a great warm-up exercise because they ease athletes into overload work and also provide a simple way to understand supination-to-pronation movement concepts.

METHOD

With the feet roughly at shoulder-width, the athlete should start with the arms at the sides, with a slight bend at the elbows and palms facing up. In a loose and athletic manner, while maintaining flexion in the elbows, swing the hands up to around the height of the neck-to-mouth area. Once the hands reach this apex, simply rotate the hands from a palm-up position to a thumbs-down position (pronate) without extending or contracting the arms. After the thumbs are turned towards the ground, simply let gravity bring the arms downwards. Do not attempt to control the weights on the way down. To repeat these steps, the athlete should use the momentum at the bottom of the swing provided by gravity to start the next rep.

2. Two-Arm Throws

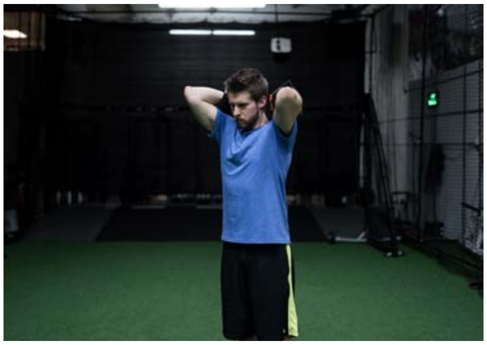

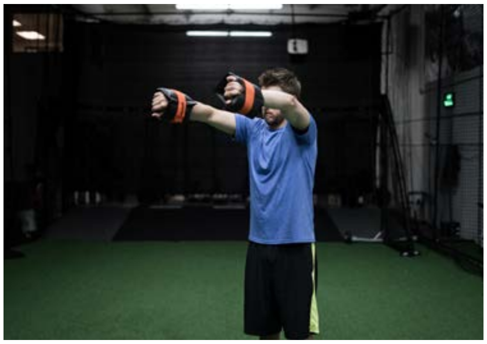

OVERVIEW

Two-Arm Throws map the coinciding events of the concentric movement of tricep extension and forearm pronation, which gives excellent feedback on how the distal end of the pitching arm can move in a straight line while rotation provides velocity. The tail end of internal rotation functions as a stress test to the posterior shoulder, making this movement a great force-acceptance exercise.

METHOD

The Two-Arm Throws wrist-weight exercise is the next progression of the Tricep Extension with Pronation movement performed with the J-Bands; the basic movement for both exercises is the same. With the feet roughly at shoulder-width apart, the athlete should start with the elbows up and the hands to the side of the ear, with the palms facing the shoulders (thumbs away from the head). Have the athlete slightly raise the chest to gain leverage to throw with. Because the goal is to create a subtle arch in the back, the language behind the phrase “slightly raise the chest” is preferred over “arch the back.” (The latter phrase creates more room for misinterpretation, creating the potential for athletes to arch their back too much, putting the lumbar spine at risk.) Once the starting position has been established, the athlete should slightly drive the chest forward to create some momentum to jumpstart the arm movement. The next step is to drive the wrist weights over the top of the elbows, simultaneously extending at the elbow (contracting the triceps) and turning the thumbs towards the ground (pronating the forearm). Athletes commonly make the mistakes of pronating their forearms towards the sky or towards the ground; the desired trajectory of forearm pronation is straight in front of the body, with pronation occurring at about eye-level for both arms. Once the forearms have pronated in front of the body, allow gravity to drop the arms so that they fall either closely by the sides or with the thumbs falling into the front of the quadriceps. To proceed to the next reps, the athlete should supinate the hands to raise them to the starting position, repeating the above steps to complete the prescribed number of reps.

3. Modified Cuban Press

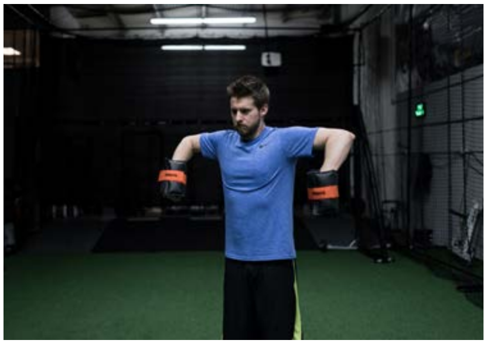

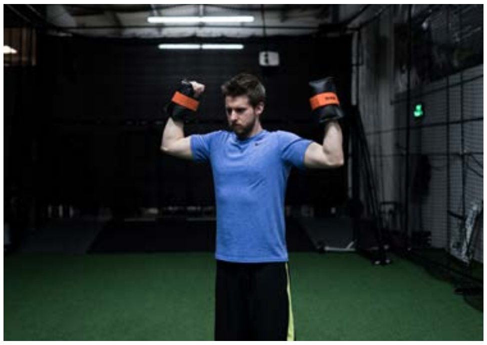

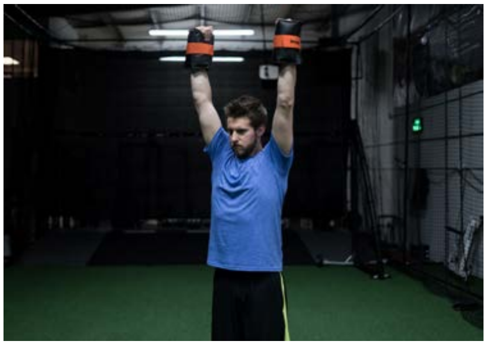

OVERVIEW

This exercise provides a great physiological stimulus due to the resistance during external and internal rotation with a light press overhead. It also passively teaches the transition by coupling external rotation with supination and internal rotation with pronation.

METHOD

Athletes should start out in a comfortable standing position, with the arms in the “inverted goalpost” position—also known as the “Flex-T” position, in which the hands hang down with the palms facing the posterior of the body (towards the butt) while the elbows are at shoulder height. While maintaining the 90-degree angle at the elbow, the next step is to rotate the forearms to the “high-cocked position” with the shoulders while simultaneously supinating the forearms (thumbs go from pointing towards the body to pointing posterior). After the forearms reach a vertical position, press the wrist weights overhead while the forearm remains in a neutral position. Once the overhead end range of motion is reached, lower the elbows back down to shoulder height with the forearms remaining vertical, then rotate forearms downward with the shoulders in a controlled manner, pronating the forearms in the process (thumbs now point towards the hips). The correct end result should have the athlete back in the original starting position. Repeat these steps for the prescribed number of reps.

4. Pivot-Pickoffs Throws

OVERVIEW

This exercise helps the athlete feel how the shoulder rotation dominates the throwing arc and allows for kinesthetic awareness as the hand drives over the elbow from a slightly supinated and externally rotated position to an internally rotated and pronated position. Like Two-Arm Throws, the tail end of internal rotation during Pivot-Pickoff Throws functions as a stress test to the posterior shoulder, making this movement a great force-acceptance exercise. While this is likely the most effective exercise in *Hacking the Kinetic Chain — Advanced Pitching* for fully grasping the concept of the correct "unwinding" of the arm, it is also the most complex exercise, creating the most room for errors. Because of this, we've divided the technique explanation of this exercise into different levels of mastery, exactly like how we explain PlyoCare drill technique in the next chapter. Visiting to our website to watch the instructional videos on this exercise before attempting is highly recommended.

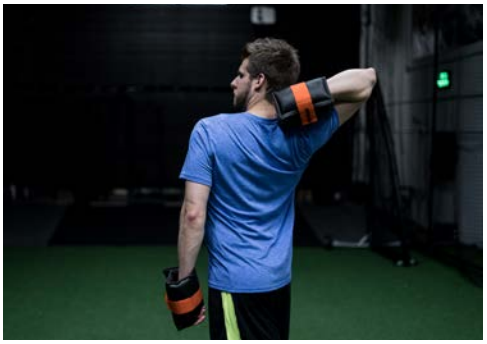

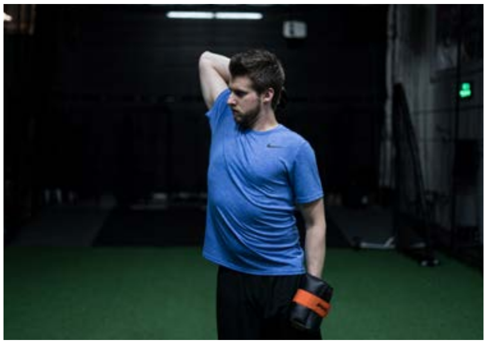

BEGINNER LEVEL METHOD

The primary goal of executing the Pivot-Pickoff Throw at a beginner level is to learn how to correctly throw the weight with the throwing side. The first step is for the athlete to establish the direction the throw will occur and then turn 90 degrees so that the throwing side faces the throwing direction. Right-handed throwers will face 90 degrees to the left in order for their right side to face the throwing direction, and left-handed throwers will face 90 degrees to the right in order for their left side to face the throwing direction. After the athlete has determined which way to face, he should stand with the feet no wider than shoulder width, with weight slightly over the balls of the feet to allow for rotation. Once the correct starting position has been established, counter-rotate the torso (and mostly likely the feet as well, depending on an individual athlete's mobility) so that the lead shoulder faces the throwing direction. In this counter-rotated position, have the throwing arm in a position where the elbow is up and the palm is directly behind the head—just like if you wanted to slap yourself in the back of the head. From there, keep the throwing hand behind the head as long as possible while the trunk rotates, causing the athlete to lead with the elbow towards the throwing direction. This step is important because a common mistake for beginners is to lead with the hand and "punch" the weight towards the target; make it clear that the goal is to utilize the trunk rotation to throw the weight. Once enough momentum is generated, the trunk will continue rotating towards the starting position, and the throwing forearm will pronate over the top of the elbow in the throwing direction, or roughly 90 degrees towards the athlete's throwing side. After internal rotation has occurred, the thumb on the throwing hand should fall into the side of the butt. The idea is to *not* follow through across the body in this exercise.

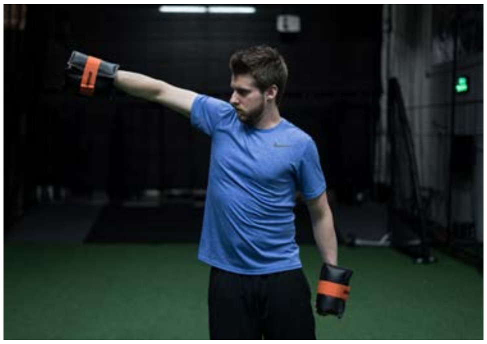

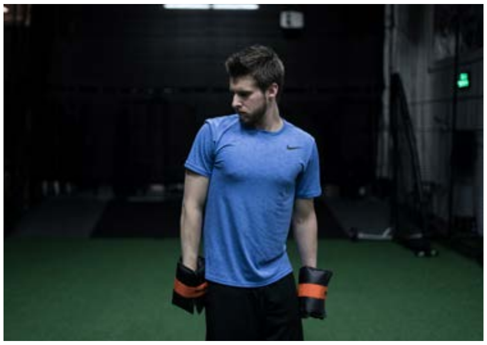

INTERMEDIATE LEVEL METHOD

The primary goal of executing the Pivot-Pickoff Throw at an intermediate level is to build upon the skills learned as a beginner by learning to correctly utilize the glove arm to leverage trunk rotation. The first step to correctly utilizing the glove arm: once the trunk counter rotation end range of motion is reached (throwing hand behind the head, elbow up), reach out with the glove hand towards the throwing direction with the forearm in a pronated position (thumb down). Do not reach too far across the body, as this will likely throw off the timing of the movement by creating a greater distance to move the 5-kg weight, defeating the purpose of this exercise. Once the glove arm has extended towards the throwing direction, the athlete should simultaneously supinate the glove hand and drive glove-arm elbow towards the back of the lead hip (similar to a kneeling cable row) while also trying to delay trunk rotation as long as possible. A key item to look for when checking the athlete's proficiency of correctly leveraging trunk rotation with the glove arm is to observe when the glove arm finishes pulling. The goal is for the glove arm to finish pulling while the throwing arm is still in layback during the Driveline Phase (positive disconnection). However, please note that the athlete should not make this a choppy movement for the sake of reaching positive disconnection. The athlete should also have noticeable lengthening of the chest ("Big Chest") right before the trunk rotates. Once the athlete has finished the glove-arm movement, allow the natural events of trunk rotation and the unwinding of the throwing arm to occur as outlined in the beginner-level section.

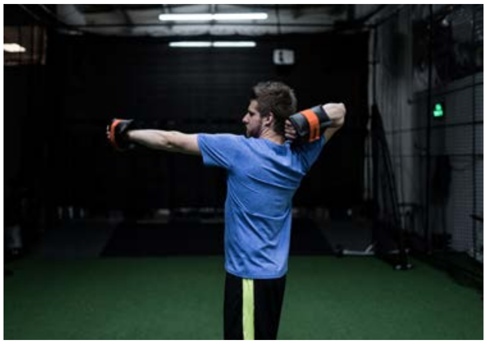

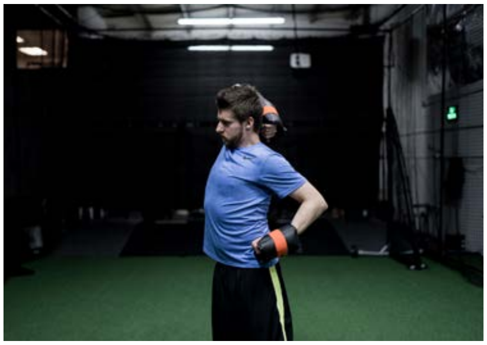

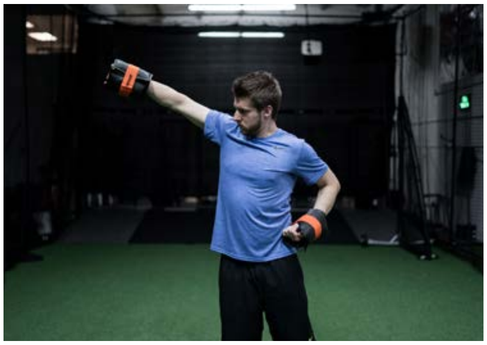

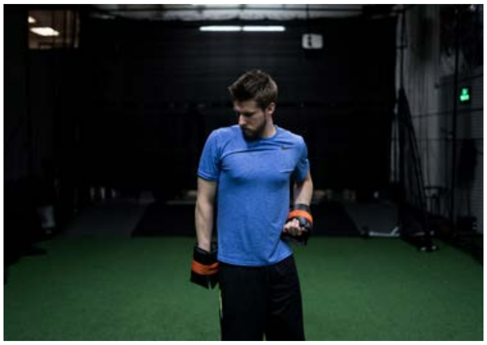

ADVANCED LEVEL METHOD

The primary goal of executing the Pivot-Pickoff Throw at an advanced level is to build upon the skills learned at the beginner and intermediate levels as well as applying the concepts of starting with more scap retraction on the throwing side and mapping the full Elbow-Spiral Phase. To further clarify the difference, the previous two levels have athletes starting with their throwing hand behind their head with the elbow up, which represents the end of the Elbow-Spiral Phase and the beginning of the Driveline Phase. The basic starting stance for advanced-level technique is the same as the previous two levels. The main difference between the intermediate and advanced levels is at the trunk counter-rotation end range of motion. Instead of pinning the palm of the throwing hand directly behind the head with the elbow up, the athlete will drop the elbow down and back to retract the throwing-side scap, causing the lead shoulder to slightly elevate, the forearm to become vertically positioned, and the throwing hand to neutralize, representing an accurate depiction of the upper half at stride-foot contact. Applying the glove-arm concepts learned at the intermediate level, the beginning of trunk rotation should cause the throwing-arm elbow to upwardly rotate into external rotation (Elbow Spiral). Once the athlete's throwing arm enters the Driveline Phase, all previously learned concepts remain the same.

Do not allow athletes to start working at an advanced level until they are executing the intermediate-level technique extremely well. Some athletes may never reach an advanced level on Pivot-Pickoff Throws, and that's ok. However, performing Pivot-Pickoff Throws at an advanced level does have a few benefits over performing the exercise at beginner and intermediate levels, which means it's a noble goal to shoot for. If athletes can perform the exercise at this level, it's a clear indication that they know how to move the upper half, making it likely that the upper half on PlyoCare drills, and eventually a pitch, should grade very highly as well. Athletes that perform this exercise at this level also tend to be much more confident with throwing a 5-kg weight attached to their arm, which means their movements will be more fluid and aggressive, creating a greater force-acceptance challenge for the posterior shoulder.

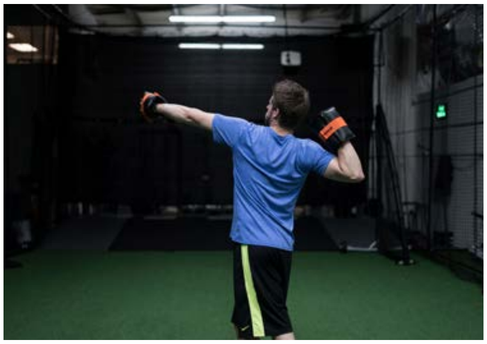

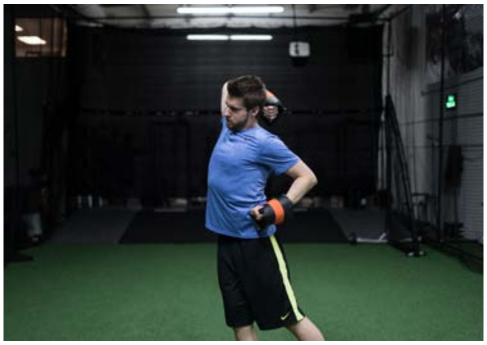

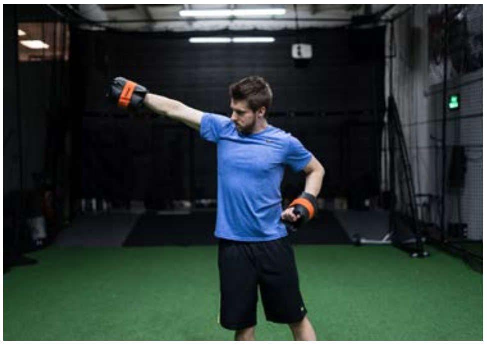

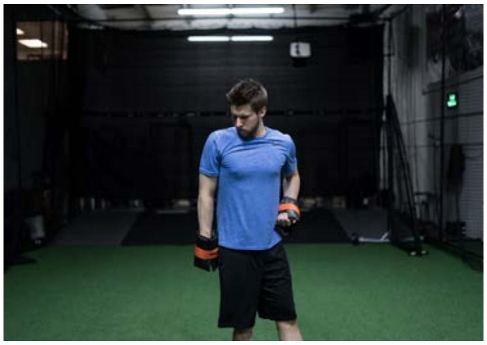

5

PLYOCARE BALL THROWING:

Building Positive Throwing Patterns

The most efficient way to remap parts of a pitching delivery is to use overloaded implements to provide better kinesthetic awareness throughout the throwing motion. There are two primary benefits of using constraint training with overload throws:

- *Developing sport-specific speed-strength by having physiological stimuli drive adaptations*
- *Reorganizing mechanical patterns through self-discovery with modification*

PlyoCare balls were designed for these purposes and for withstanding repeated high-intensity throws into hard surfaces, such as concrete walls and heavy mats. These soft-covered, sand-filled balls serve multiple purposes in our throwing programs, which makes them versatile and important tools. When throwing PlyoCare balls, establish a perceived throwing-target level around mid-chest to eye level, an external cue that can help optimize the brain's ability to correctly self-organize the body's throwing patterns.

By constantly testing and retesting the most efficient throwing patterns with the PlyoCare balls, we are able to provide a list of extremely effective drills that provide positive-mechanical feedback for a pitcher, as well as maximize the ability to produce

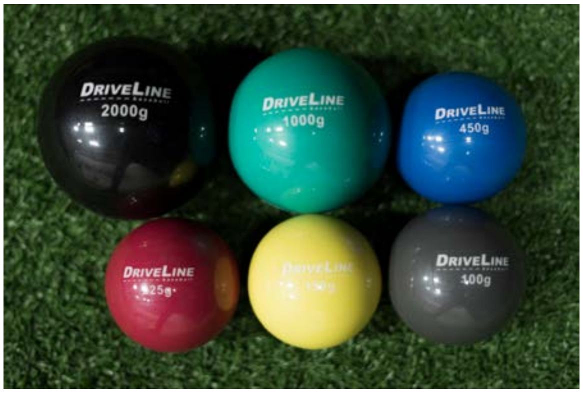

and accept force. Our PlyoCare drills are categorized into standard and specialized drills. Our standard PlyoCare drills are designed to cover the majority of common throwing-pattern deficiencies that we see in athletes, making up the bulk of an athlete's throwing workload each day. Specialized PlyoCare drills are designed to correct more specific throwing-pattern deficiencies that the standard PlyoCare drills may not target enough, and they are used in our Specialized Movement Focus throwing programs.

This chapter provides an overview and covers the proper execution of each PlyoCare drill that exists to date. For some of the more technical drills, the techniques will be broken down into beginner, intermediate, and advanced levels. Here's a breakdown of how we perceive each level:

BEGINNER LEVEL

The expected technique for a first-time athlete. This level of performance is mildly acceptable for the time being, but it needs to advance in order for a better quality of training.

INTERMEDIATE LEVEL

An acceptable level of performance. Some athletes might never advance out of this category, which is fine. Performing drills at this level is sufficient enough to self-organize throwing patterns in a positive manner.

ADVANCED LEVEL

An elite level of performance, and a clear goal to shoot for. Most athletes that fall into this category are professional or high-level college athletes that combine very good general movement quality with great understanding of the drill.

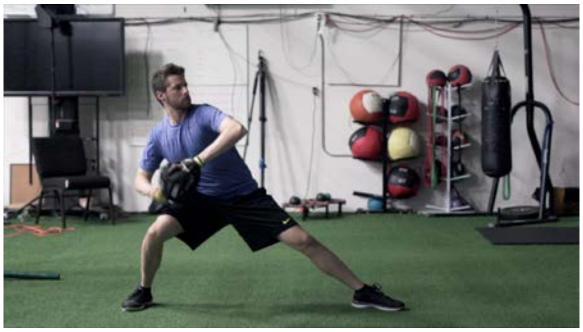

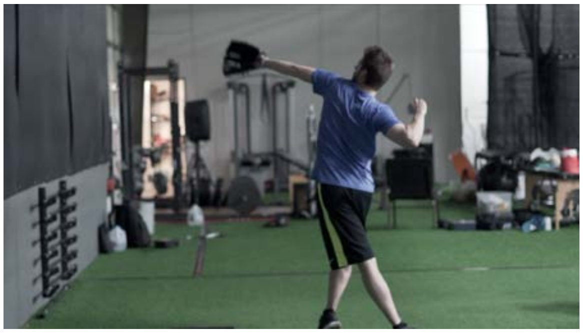

Reverse Throws

CLASSIFICATION
Overload Standard PlyoCare Drill

DRILL PURPOSE
Reverse throws are designed to train the posterior shoulder by activating the muscle group during the initial stages of the trunk counter-rotation, as well as providing an active stretch of the anterior shoulder muscle group during the follow through. Backwards chaining principles are also featured during this movement due to the focus on mastering thoracic-spine rotation during counter-rotation, which is an important component for high-velocity throws. Reverse Throws are also a good ballistic exercise that provides a strong psychological stimulus to not only provide power but also a conscious awareness of how an athlete's intent is focused on maximizing trunk-rotation speed in order to increase ball velocity.

COMMON MISTAKES
When executing Reverse Throws, there are three common mistakes to look out for when athletes first attempt this drill:

Starting Counter-Rotation from a Static Position
It's difficult to perform any explosive movement from a static position. Often, athletes will try to start their Reverse Throw from a still position with the ball on the ground. This makes it difficult to maximize speed and athleticism during the exercise. To correct this, we suggest having the athlete start in an upright position and then reach downward towards the ground to create some fluidity and tempo with the movement.

Collapsing the Lead Leg
During trunk counter-rotation, many athletes will let the lead foot roll over and the lead leg collapse, essentially causing them to mostly rotate at the hips. As stated before, one of the goals of the Reverse Throw is to rotate with the thoracic spine, which is trained less effectively when the lead foot rolls over. To correct this, keep the lead foot solidly planted on the ground while opening the chest to the throwing dominant side.

Lawnmower Pull/"Option Pass"
Sometimes at the point in time when athletes begin to make the first move to rotate and throw the ball, instead of making the initial move of starting the trunk's counter-rotation first, they begin to pull the ball towards the body by retracting the throwing-arm elbow, similar to starting a lawnmower. Once the trunk rotation has started, the athlete will then oftentimes push the palm of the hand at the wall, similar to throwing an option pass in football. This prevents the thrower from understanding the kinesthetic awareness of how the events of thoracic spine counter-rotation and a delayed throwing-arm maximize power and ball velocity. To correct this, help the athlete try to keep the throwing arm fixed to the shoulder as the trunk counter-rotation leads the movement and then drive the back of the hand at the wall.

EXECUTING THE REVERSE THROW

Beginner Level Method
A beginner-level Reverse Throw is what to expect from a first-time athlete. The first step is to kneel down on the throwing-side knee. An athlete that is able to execute a Reverse Throw at a beginner level is able to perform the movements of opening the chest towards the throwing dominant side and throwing the ball into the wall.

Intermediate Level Method
To build upon what the athlete learned at the beginner level, the ability to execute a Reverse Throw at an intermediate level is highlighted by performing the movements of opening the chest to the throwing-dominant side while simultaneously keeping the lead foot locked onto the ground. The athlete should keep the throwing arm fixed to the shoulder during trunk counter-rotation and drive the back of the hand at the wall. The throw should result in a ball flight that targets the wall right behind the athlete's head.

Advanced Level Method
The mark of an advanced-level Reverse Throw is what we view as executing the kinematics at a highly technical level with both speed and power. To optimize the starting position, kneel down on the throwing side with the lead leg straight in front at an approximately 100 to 115-degree angle, with the lead foot slightly internally rotated (much like the lead foot's positioning in a Rocker Throw). Once the starting position has been es-

tablished, the athlete should start in a gathered position with the trunk in a vertical position. Have the athlete reach towards the ground with the throwing arm at an up-tempo pace, and once the ball touches the ground, aggressively counter-rotate and drive the back of the hand towards the wall all while the lead foot maintains solid contact with the ground. There should be a slight amount of hip rotation, but the goal should be to maximize thoracic-spine mobility during the counter-rotation. Catch the ball off the wall, and proceed with the next rep at an up-tempo pace and repeat for the suggested amount of reps in the program.

Pivot-Pickoff Throws

CLASSIFICATION
Overload Standard PlyoCare Drill

DRILL PURPOSE
The most constrained, and also the most complex, drill to master, Pivot-Pickoff Throws are designed to accomplish several tasks. From a throwing-kinematics standpoint, this drill is designed to take out the lower half entirely in order to isolate the upper half in a position similar to its position at stride-foot contact during a pitch. This forces the athlete to learn how to properly create positive disconnection. Because we isolate the upper half and use much heavier implements than a baseball with this drill, it functions as a great modality to train the Elbow-Spiral and Driveline Phases as well. From an anatomical standpoint, Pivot-Pickoff Throws train internal-rotator and elbow-extensor strength, shoulder mobility, and forearm pronator-flexor dynamic strength. The upper half contains the most moving parts, so trying to execute this drill at an advanced level is an important focus for most athletes.

COMMON MISTAKES
Because Pivot-Pickoff Throws are the most complex drill out of all PlyoCare drills, there are a lot of mistakes that can occur, so you may need to refer to the Throwing Mechanics chapter to properly evaluate an athlete's technique. From our experience training athletes, here are a few common mistakes we see with this drill:

Throwing Breaking Pitches
Many athletes have poor awareness of forearm positioning, which can be exacerbated when throwing 1- and 2-kg balls. Sometimes athletes begin with too much supination in the throwing forearm, causing them to hold that position too long during the Driveline Phase, resulting in a curveball or cutter/slider spin axis. To correct this, make sure the athlete has the ball facing the same direction as the chest (neutral, right-hander towards third base, left-hander towards first base) at the moment when the forearm is vertical at the simulated stride-foot contact position, not when facing the athlete's head. Other times athletes just try to be a hero by grabbing the ball with two fingers, which often results in a slider spin axis. Unless an athlete has abnormally large hands and/or abnormally high grip strength, it's likely optimal to use three fingers when gripping a 1- or 2-kg ball. One internal cue that often helps correct this without disrupting an athlete's intent is "feel the ball come off the ring finger when it leaves the hand." In reality it won't actually come off the ring finger like a circle changeup, but this feel is usually an indicator that the athlete has turned his hand over in time during internal rotation.

Lack of Trunk Counter-Rotation
Athletes commonly keep their feet stuck in place instead of rotating them to allow the lead shoulder to close off. Throwing from a partially open position is better than nothing, but it only partially maps positive disconnection (see more in the Lack of Positive Disconnection section below) and the Driveline Phase. To correct this, two simple internal cues that often work to close off the trunk without disrupting an athlete's intent are to either "crank the throwing-side lat down to the back of the throwing-side hip" or "slightly elevate the lead shoulder at the target."

Pushing the Ball
Some athletes might push the ball a little bit at first, but don't be too quick to change their throwing-side mechanics, especially if they're performing most of these steps correctly. With that said, there are often two ways athletes push a ball, and knowing the difference is important. The first way is whether or not the athlete leads with the hand before the elbow, very much like in a shotput-type fashion. These athletes will often exhibit the same behavior on wrist weights as well, and internally cueing them to feel some layback by leading with the elbow isn't a bad idea.

However, some athletes actually perform the outlined steps correctly and simply don't generate much layback, which gets misinterpreted by many observers as pushing the ball. Sometimes it takes a little while for the athlete to trust the drill and/or weighted balls, and sometimes it can be due to a mobility constraint, so knowing their screening results is a key piece of information in these instances. Show patience with these types of athletes and allow the PlyoCare balls to do their job over time when possible.

Lack of Positive Disconnection

There are a few common reasons why athletes fail to create positive disconnection, and you actually have to check the throwing and glove sides to correctly identify the reason. On the glove side, the most common problem athletes encounter is pulling the glove arm at the same time as the trunk rotation. There are three common reasons for this issue:

1. *Reaching too far across the body with the glove arm, creating too far of a distance to travel*
2. *Keeping the glove-arm forearm too close to the body ("chicken wing"), not creating enough length to feel how to properly move the glove-arm elbow through space*
3. *Pulling the glove arm dependently of the lead shoulder, not independently of the lead shoulder*

An often-overlooked reason that athletes commonly lack ideal positive disconnection is because they don't create enough scap retraction on the throwing side, which often occurs due to a lack of trunk counter-rotation. Correcting positive-disconnection issues is usually the most difficult part of optimizing the Pivot-Pickoff Throw, so please reading the section below on how to properly execute this drill in order to learn how to correct this issue.

EXECUTING THE PIVOT-PICKOFF THROW

Beginner Level Method

If athletes have performed wrist weights before, then they'll understand the basic Pivot-Pickoff Throw stance. For those that have not read the wrist-weights section, the first step is for the athlete to establish the direction the throw will occur and then turn 90 degrees so that their throwing side faces the throwing direction. Right-handed throwers face 90 degrees to the left in order for their right side to face the throwing direction; left-handed throwers face 90 degrees to the right in order for their left side to face the throwing direction. After the athlete has determined which way to face, he should stand with the feet no wider than shoulder width, with his weight slightly over the balls of the feet to allow for rotation. Once this starting position is established, executing a typical beginner-level Pivot-Pickoff Throw means simply counter-rotating the torso and throwing the ball at the wall or target.

Intermediate Level Method

Once the basic Pivot-Pickoff Throw movement has been established at a beginner level, the goal for executing this drill at an intermediate level is primarily optimizing the throwing side and, secondarily, gaining some understanding of how to properly use the glove arm. The first step to this process is making sure the athlete achieves sufficient counter-rotation of the torso. Sufficient counter-rotation means that the athlete's lead shoulder is slightly elevated and pointing at the wall or target, and the throwing-side lat is cranked slightly down towards the back of the throwing-side hip. The goal behind this is to slot athletes into a pre-set position where they simulate being at max-scap retraction on their throwing side at stride-foot contact. After sufficient counter-rotation has been achieved, make sure the athlete's forearm is in a neutral position (ball facing same direction as the chest) and somewhat vertical. Next, the athlete should reach out towards the wall with the glove arm in a pronated position, like for wrist-weight Pivot-Pickoff Throws. After that, simply pull the glove arm and throw the ball. Check to make sure the PlyoCare ball's spin axis resembles that of a fastball, not a breaking ball. When the athlete gets comfortable with the Intermediate level movements, they should try to move as fluidly as possible at the prescribed RPE for the day.

Advanced Level Method

Performing Pivot-Pickoff Throws at an advanced level is an extremely technical movement where every small detail matters. With a technical movement comes a technical explanation, so let's assume the athlete is executing the steps of the intermediate-level method very well. The last layer to add is the targeted training of positive disconnection. To begin our explanation of this drill at an advanced level, let's start at the point where the athlete is at counter-rotation (lead shoulder elevated,

forearm neutral, glove arm extended towards the wall/target in a pronated manner, etc.). Before the athlete rotates to throw, the first move should be to supinate the glove hand and drive the glove-arm elbow towards the back of the lead hip. The athlete should attempt to delay trunk rotation as long as possible by keeping the lead shoulder closed while the elbow drives towards the back of the lead hip. Like referenced in the wrist-weights section, this movement should resemble that of a kneeling cable row. In other words, the glove arm should pull independently of the lead shoulder, not dependently of the lead shoulder. Once the glove arm starts to travel through space ahead of the initial trunk-rotation movement, the athlete should naturally unwind the trunk to throw the ball as outlined in the beginner- and intermediate-level sections.

Related note: If athletes need additional help with feeling the glove arm in space, placing a 450-g or 1-kg ball in their glove hand can improve proprioception. There's also nothing wrong with having athletes use their glove. However, placing anything heavier than a 1-kg ball in their glove hand (most commonly a 2-kg ball) is *not* recommended because many athletes are not strong enough to move that type of weight quickly enough, which can have an adverse effect on training correct glove-arm movement.

These steps are helpful to know how to perform the drill, but how do you truly know if an athlete has executed the Pivot-Pickoff Throw at an advanced level? There are a few signs to look for. First, as trunk rotation begins, you should notice that the athletes that create elite positive disconnection have an apparent lengthening in the chest ("big chest") due to the scap retraction created on both the glove and throwing sides. This should then cause trunk flexion towards the wall/target, creating a natural path to forwardly rotate the throwing shoulder during the Driveline Phase. This should cause a visual weight shift to the throwing side after the throw has been completed. Athletes that create poor positive disconnection tend to keep their weight centered between their feet throughout the throw, even moving away from the wall/target in some instances. We strongly suggest checking out our videos outlining Pivot-Pickoff Throws on our website to get a better visual in order to optimize how well you can apply this information to training your athletes.

Roll-In Throws

CLASSIFICATION
Overload Standard PlyoCare Drill

DRILL PURPOSE
If the Pivot-Pickoff Throw is designed to isolate the upper-half kinematics, then Roll-In Throws represent the next progression by adding a linear component. The drill minimizes the lower-half rotation component, keeping the task of drilling trunk stacking and lead-leg blocking, two of the most important variables to master for high-velocity throwing, as simple as possible. This drill is nowhere near as technical as the Pivot-Pickoff Throw, so athletes usually pick up on the technique quickly.

COMMON MISTAKES
While Roll-In Throws are less complex than Pivot-Pickoff Throws, there are two common, but easily correctable, mistakes that athletes make while attempting this drill:

Actively Planting Sideways

The most common mistake to watch out for is athletes' actively planting their bodies sideways (evident by planting the back foot sideways) when they are about to throw. Basically, an athlete should stay totally linear towards the wall in this drill. (See a full explanation in the "Executing the Roll-In Throw" section.) A good internal cue that helps fix this is to tell the athlete to "throw on a walk" and relate it to how middle infielders throw on the run.

Along these same lines, coaches and trainers often mistake two different events, actively planting the back foot sideways and back-foot counter-rotation after contact with the ground, as one flaw. To be completely clear, actively planting the back foot sideways before the throw means the athlete is not executing the drill correctly, often representing a break in momentum. However, back-foot counter-rotation after contact with the ground is completely normal. Unless the athlete is extremely mobile, the back foot is going to counter-rotate due to the following chain of events: As the center of mass continues towards the wall, the arm action going through the Pickup Phase causes scap retraction on the throwing side, resulting in

counter-rotation of the torso in turn causing slight counter-rotation of the pelvis, thus creating counter-rotation of the back foot. How much counter-rotation each body part goes through during the Roll-In Throw depends on each athlete's mobility.

Loss of Trunk Stack into SFC

Athletes commonly lose their trunk stack as the stride foot makes contact with the ground. While sometimes this is due to an arm-action deficiency or a mobility constraint, it is often just a general movement-pattern deficiency resulting from trying to throw everything low for an extended period of time. Simply reinforcing the correct target level on the wall and reminding the athlete to keep the chest up as long as possible into stride-foot contact are usually effective strategies for correcting this mistake.

Side note: Lead-leg blocking deficiencies are also common with this drill, but we address this issue in the explanation of Rocker Throws since they are the primary drill to fix this issue.

EXECUTING THE ROLL-IN THROW

Beginner Level Method

Executing this drill at a beginner level is fairly easy. The first step is for the athlete to face the target, standing comfortably with the throwing hand holding the ball in a neutral position (palm facing the hip, thumb up) slightly in front of and at waist level. Once this starting position has been established, simply walk towards the wall/target (distance of the walk may depend on how much space the athlete has to work with), make sure to not actively plant the back foot sideways, step with the normal stride leg, and throw the ball. If the athlete's trunk does not close off all the way, this is fine. Emphasize the linear component with this drill.

Intermediate Level Method

Once the basic Roll-In Throw movement has been established at a beginner level, the primary goal for executing this drill at an intermediate level is to help the athlete optimize trunk stacking. Assuming all of the steps from the beginner level are followed, have the athlete keep the chest up as much as possible into foot strike. Trunk stacking can be subjective, so to make it simple: if the athlete's shoulders are fairly level and the throwing-side scap appears pinned back at stride-foot contact, then the athlete has sufficiently kept the trunk stacked.

Advanced Level Method

Performing Roll-In Throws at an advanced Level is mostly a combination of elite upper-half movements at high intensities and executing the intermediate level steps very well. To keep it simple, if an athlete displays great arm action, trunk stack, positive disconnection, lead-leg blocking, and does not show any signs of common mistakes, then the athlete is likely performing this drill at an advanced level.

Coach's Note: Athletes and coaches are often confused about how we approach arm action for this drill, mostly regarding a short- or long-arm action. Let's address this briefly. If an athlete uses his full-arm action for Roll-In Throws, and the arm action has nothing wrong with it, does it really need to be changed for the purpose of the drill? Again, the Roll-In Throw is designed to drill trunk stacking and lead-leg blocking, so as long as the arm action isn't bad, we usually leave it alone. If you notice that an athlete's arm action has become inefficient during this drill, then it's wise to step in and have the athlete keep the arm action compact as the center of mass travels towards the wall/target. If you need ideas for helping an athlete keep his arm action compact on this drill, then read the Scap-Retraction Throws section for an explanation on optimizing the Pickup Phase.

Rocker Throws

CLASSIFICATION

Varied Standard PlyoCare Drill

DRILL PURPOSE

Rocker Throws help athletes isolate the lead-leg blocking movement pattern by removing the stride from the equation. This drill also should help the athlete self-organize an optimal trunk-rotation pattern, which is somewhat mutually inclusive with feeling the lead-leg blocking movement pattern.

COMMON MISTAKES
When athletes are executing Rocker Throws, here are three common mistakes to look for:

Incorrect Stance
We cover how to start with a correct stance in the Method section below, but look out for these common mistakes regarding stance:

1. *Too narrow of a base*
2. *Lead foot facing the wrong direction*
3. *Center of gravity is too high*

An athlete's starting with too narrow of a base can hinder his ability to properly block with the lead leg due to the lead foot's close proximity to the center of mass. More distance is not necessarily better, but the lead foot should be far enough away from the torso to create an appropriate lead-leg blocking angle to stop the center of mass's linear momentum. If you observe athletes flying over the top of the lead leg when they throw, chances are good that they have too narrow of a base. If an athlete starts out with the lead foot facing the wrong direction, he may not get an accurate feel for how the lead leg works in a throw, which minimizes the odds of the newly learned movement patterns of transferring to a pitch or regular throw. Athletes that start with too high of a center of gravity sometimes have too narrow of a base to start out with, but sometimes it's as simple as accidentally having their legs locked out. A quick solution is to have them sink their hips a little to gain some flexion in the legs so they can shift their weight with the lower half.

Lack of a Lead Leg Block
We mentioned in the Roll-In Throws section that lead-leg blocking deficiencies are common in many athletes during PlyoCare drills. This is the primary drill where we seek to fix lead-leg blocking issues. If athletes are having trouble properly utilizing the lead leg, check for the following issues:

1. *Too narrow of a base*
2. *Hamstring-flexibility issues*
3. *Hip-mobility issues*

As mentioned before, having too narrow of a base can minimize the lead leg's effectiveness in stopping the center of mass's linear momentum. For points two and three, refer back to the athlete's mobility screen to check for these. If the athlete fits somewhere in points two and/or three, then the athlete's lead-leg movement pattern may clean up over time through continued execution of corrective exercises. If athletes fit none of the three points, then they likely just have trouble feeling the movement pattern. In these cases, it's up to the coach's discretion on how help each individual athlete feels the correct lead-leg blocking movement pattern.

Leaving the Back Foot on the Ground
We've seen plenty of athletes try to leave the back foot on the ground during their first attempts at performing Rocker Throws. The general idea of Rocker Throws is not unique to Driveline; many athletes have gone through versions of this drill in which they are instructed to leave the back foot on the ground. The internal cue of "finish the pitch" typically solves this issue very quickly without affecting the athlete's intent.

EXECUTING THE ROCKER THROW

Method
The Rocker Throw is not a complex drill, so breaking it down into different levels of mastery is not necessary. The first step is to establish a solid starting base, which is the most technical part of the drill. To start out, the athlete's back foot should be parallel with the rubber, the lead foot very slightly internally rotated to face the wall/target (realistic landing position). Make sure the lead foot is far enough away from the center of mass to create a good lead-leg blocking angle, which may understandably take some trial and error to figure out. Once the ideal base width is established, lower the center of gravity a little (sink the hips) to create flexion in the knees to allow for lower-half movement.

After the center of gravity is slightly lowered, the athlete is ready to proceed with the remainder of the drill. While keeping the upper half stacked, simply shift the weight forward with the lead leg, then shift the weight to the back leg, then explode off of the back leg to supply some linear momentum, and throw the ball. When exploding off of the back leg, try to drive the center of mass towards the wall/target (X plane), not straight up towards the sky (Z plane). Use the lead leg to put force in the ground towards the target (X plane) to stabilize the lead leg while the throwing shoulder rotates forward towards the wall/target, which should help train the lead-leg blocking mechanism.

Walking-Windup Throws

CLASSIFICATION
Varied Standard PlyoCare Drill

DRILL PURPOSE
Walking-Windup Throws are purposely the least constrained and least complex PlyoCare drill. They simply teach athletes to optimize their athleticism and intent during a pitch. From a more technical perspective, the Walking-Windup Throw combines the lower-half rotation component with the linear component learned with the Roll-In Throw. This drill is essentially a progression from Roll-Ins, though we have athletes perform them after Rocker Throws to blend the correct lead-leg blocking and trunk-rotation patterns into a throw that closely mimics the pitching delivery. This drill is done with a quick pace to minimize wasted movement and maximize kinetic-sequencing efficiency.

COMMON MISTAKES
The least technical drill of the bunch, there are only two common ways athletes tend to make mistakes with Walking-Windup Throws. This drill is meant to blend the throwing patterns learned from the other PlyoCare drills into an athletic throwing movement, so most throwing-pattern deficiencies will be targeted in other drills.

Stopping Momentum at Max Leg Lift
The word "windup" within the drill name is often mistaken by athletes as a cue to take their time and stop momentum at max leg lift. While we cover the more technical explanation of how to execute this drill below, athletes should actually perform this drill with a high sense of urgency. Here's an effective way to communicate this to them: have them envision the sense of urgency they would use to pitch with if Billy Hamilton (or insert another serious base-stealing threat) was on first base, and they know he's about to steal second base. They're likely to minimize their time to home plate, right? Have them apply that kind of sense of urgency to Walking-Windup Throws.

At the same time, this drill does not specifically teach the concept of "drift." How each athlete projects his center of mass towards the target at max left lift varies between athletes. Allow them to play around with the drill to find the right feel for them. Feedback from radar guns can also play a key role in this self-discovery process.

Step-Behind Technique
We always emphasize being as athletic as possible within this drill, so sometimes athletes take this cue a step too far and use a Step-Behind Technique, similar to the footwork they would use on a Pulldown (more on technique in the Pulldowns chapter). To correct this, simply follow the steps outlined below.

EXECUTING THE WALKING-WINDUP THROW
Method
The first step to correctly executing a Walking-Windup Throw is to establish the target and to establish a throwing lane (create an imaginary straight line) towards that target. Once the throwing lane has been established, take a couple of steps off to the glove side, exactly like a field-goal kicker does in football. When the athlete gets to this starting position, have them walk in an upbeat fashion towards the throwing lane at an angle. The distance this walk covers depends on the amount of space available, as well as what each athlete feels comfortable with. When the athlete is ready to make his throwing movement and has reached his throwing lane, he should plant the back foot, lift the lead leg, and redirect his momentum towards the target. Some athletes use a full leg lift, whereas others only use an abbreviated leg lift, or even a slide step. There's no right or wrong way to lift the lead leg as long as it helps optimize the athlete's ability to perform the rest of the drill correctly. After momentum has been redirected, throw the ball as athletically as possible.

We are commonly asked questions about why we have athletes walk in at an angle and not straight on towards the target. Have you ever tried to maintain your momentum towards the target while also turning a full 90 degrees and lifting your lead leg to max leg lift at the same time? It's nearly impossible. Walking straight to the target contributes to athletes' stalling their momentum, so we adjusted the drill to walk in at an angle so athletes would only have to turn their bodies about 45 degrees, making it much easier to maintain momentum and optimize athleticism throughout the drill.

Scap-Retraction Throws

CLASSIFICATION
Overload Specialized PlyoCare Drill

DRILL PURPOSE
Scap-Retraction Throws are specifically designed for athletes that have an arm action dominated by the throwing hand's traveling towards 2nd base. None of the standard PlyoCare drills cover this particular arm-action deficiency, so we implemented this drill to help athletes feel how the elbow movement dominates the arm action during the Pickup Phase. The ultimate goal for this drill is to take steps towards creating a Pickup Phase that helps retract the athletes' throwing-side scap to maintain trunk stack into stride-foot contact and to optimize their ability to create positive disconnection. This drill is usually performed between Pivot-Pickoff Throws and Roll-In Throws. Performing Scap-Retraction Throws after Pivot-Pickoff Throws helps blend Elbow-Spiral and Driveline Phase concepts into a more complete arm action, and performing this drill before Roll-In Throws helps blend a more ideal arm action with a throw containing a linear component.

IDENTIFYING THE RIGHT CANDIDATES
Without question, determining which athletes are candidates for this drill is subjective, but we provide descriptions here to help you make the most educated programming decisions.

Rule #1: Always look at the effect on the Elbow-Spiral Phase to determine if the athlete's Pickup Phase needs to be adjusted. Here's a list of issues we look for when considering implementing this drill into an athlete's routine:

Reaching Towards 2nd Base
Some athletes have an arm action that is clearly dominated by the throwing hand's traveling towards second base while the center of mass is traveling towards home plate. If the athlete's throwing arm tends to extend towards second base when separating the hands and in turn creates an inability to roll into a good Elbow-Spiral Phase, then Scap-Retraction Throws are a great way to target this deficiency.

Coach's Note: Don't confuse this deficiency for the athletes' forearm appearing to point towards 2nd base a little bit when their throwing-side scap retracts during a clean Pickup Phase. Refer back to Rule #1.

Pronated Takeaway
If an athlete has a very pronated throwing hand when the hands separate, odds are he also has excessive elbow climb that creates excessive shoulder abduction (leading to the Inverted W position at stride-foot contact), assuming this pronated position is maintained during the Pickup Phase. This drill helps neutralize the throwing hand during the Pickup Phase, mapping a cleaner arm path by making scap retraction have a more prominent role during the Pickup Phase.

Immediate Elbow/Forearm Elevation
We don't see this problem much in person at our facility, but we do acknowledge that it exists. Some athletes immediately take their forearm and elbow upwards at the same time as soon as the hands separate. This typically leads to early trunk rotation since the throwing-side scap minimally retracts, creating nothing to hold the shoulders closed as the glove arm begins to clear. This deficiency can even have an adverse effect on how the lower half works, often creating the need to land early since the throwing arm is in a position to go into the Elbow-Spiral Phase too soon. This can lead to creating a less than ideal lead-leg blocking angle, as well as minimal hip/shoulder separation because the hips have minimal time to rotate. Implementing this drill correctly can be the first step to addressing several problems at once.

EXECUTING THE SCAP RETRACTION THROW
Method
The beauty in Scap-Retraction Throws is that they are incredibly easy to perform. They're understandably awkward at first for any athlete with a major arm-action issue, but the technique is very easy. First, establish the location of the wall/target and turn in a position so that the glove side is facing it, much like pitching from the stretch. Hold the ball in a neutral position with the throwing hand (palm facing the hip, thumb up) slightly in front of the body at waist level, exactly like the preset position for a Roll-In Throw. After the starting position has been established, the athlete should approach the throw

like he's playing catch with the wall/target. To break this drill down in a more technical manner, when the athlete takes a small stride towards the target, the first move to make with the throwing arm should be to take the elbow in a posterior direction (like a single arm row) instead of letting the hand/forearm travel towards 2nd base. Once the stride and first move with the throwing arm is made, simply throw the ball. While this may appear choppy at first for some athletes, the eventual end product to shoot for is a fluid movement that improves the efficiency of the athlete's arm action.

Step-Back Throws

CLASSIFICATION
Varied Specialized PlyoCare Drill

DRILL PURPOSE
Step-Back Throws were designed to optimize how athletes transition their center of mass down the mound by reorganizing the lower-half kinematics. No, this drill is *not* for teaching the athlete how to "push off" with the back leg... sort of. We discussed in the Lower-Half Pitching Mechanics chapter the main roles of the back leg:

1. *It is not the sole driving force, but it does aid the linear movement of the athlete's center of mass down the mound and towards the target. This means the speed of transition, distance covered, etc. This plays a direct role in setting a lead-leg blocking angle and the amount of time the hips have to rotate*
2. *Weight distribution and timing of when the back foot disengages from the rubber plays a role in an ability to maintain trunk stack*
3. *While mobility also plays a role, the back-leg movement pattern can create a counterbalance mechanism that aids in the role of creating anterior pelvic tilt when the hips rotate. This clears space for trunk flexion towards the target*

This drill is a replacement for Walking-Windup Throws for athletes that have already mastered throwing as athletically as possible, but who need to learn how to maximize their lower-half efficiency. Step-Back Throws overload the linear component by making the athlete reverse direction out of a slightly exaggerated amount of back leg flexion. This drill also helps athletes figure out their back-foot weight distribution, as well as help them find their own ideal timing for when to release the back-foot anchor.

IDENTIFYING THE RIGHT CANDIDATES
This drill should be implemented much more sparingly than Scap-Retraction Throws because perfecting lower-half movement patterns, particularly the kinematics during the transition of the center of mass towards the target, should be saved as the absolute last thing on the agenda. Mastering the Standard PlyoCare drills, the intent to throw gas, and maximizing upper-half mechanical efficiency should all come first before trying to eke out that last little bit of velocity from tweaking the lower half. If an athlete has mastered those three areas and you suspect that his lower half can still improve, here are some signs that Step-Back Throws may be a welcome replacement for Walking-Windup Throws:

Early Release of Back Foot Anchor
To be fair, it's virtually impossible to quantify this deficiency, especially when it's not terribly obvious. However, there are a few signs that show the back-foot anchor releasing from the ground too early:

- *Minimal amount of trunk flexion during the Driveline Phase. This will also be related to how much anterior pelvic tilt is created when the hips rotate.*
- *Back knee pulls very close to the lead leg, making the back-leg femur appear completely vertical. You may notice in more extreme cases where the back knee pulls so close to the lead leg that the femur goes past vertical.*
- *Back foot pulls high off of the ground during the early stages of trunk rotation.*

The three signs listed above are generally related to one another, and we've listed them in order if you're thinking in terms of backwards chaining. That means if one reverse engineers the listed order, the back foot's pulling off of the ground early creates the ability for the athlete to pull the back knee close to the lead leg, minimizing the counterbalance the back leg creates, thus minimizing anterior pelvic tilt and trunk flexion. See, isn't this fun? The description of how to perform the drill will help move athletes towards fixing this issue.

Upward Direction of Hip Extension

Some athletes have the tendency to extend their back leg, and also the back hip, in an upward direction, rather than towards the target. It's important to reiterate that we are not advocates of striding as far as possible, but if an athlete's stride seems like it's too short, then upward hip extension may be the culprit. While this can be a sign of a breakdown of the linear component, it also can mean that the athlete probably understands the general concept of how to use the back leg, but needs to adjust his weight distribution and understanding of back-foot anchoring.

EXECUTING THE STEP-BACK THROW

Step-Back Throws are easy to execute in principle, but fairly technical to explain. To perform this drill, face the body in a manner so the glove side is pointing at the target. Once the starting position has been established, take a small step (roughly 2 feet or so in length) away from the target with the back foot at a medium tempo. The athlete should begin to lower the center of gravity to create flexion in the back leg and the hips as the center of mass transitions onto the back leg. An appropriate internal cue might be to have the athlete envision "sitting in a chair" during this movement. Once the center of mass has transitioned to the back leg, the athlete should slightly lift the lead leg and let it slightly cross over the back leg so that the lead foot is on the 2nd base side of the back foot. This is the perfect time to establish the correct weight distribution on the back foot—on the middle to outside of the foot.

When the athlete has totally stopped his momentum going towards 2nd base, the first move should be to aggressively swing the lead leg towards the target to jumpstart the center of mass transition. While the lead leg swings towards the target, the athlete should simultaneously extend the back leg, having the feeling of "spreading the floor" with the back foot. This is *not* to create a goal of applying as much force as possible; it's just to help athletes apply force with the back foot away from the target (X plane) instead of upward (Z plane). The goal should be to maintain weight distribution on the back foot, keeping it anchored to the ground as long as possible while the center of mass is moving towards the target. When the lead leg begins to make its move into landing, the hips will begin to open and the back foot will pivot accordingly without any conscious focus. As the lower half unwinds, the athlete should execute upper-half throwing mechanics as normal.

It's typical for this drill to be a little awkward for athletes to execute when first trying it, particularly with moving their center of mass out of an amount of back leg flexion that they are not accustomed to. For athletes first beginning this drill, create a primary focus of mastering their weight distribution and back-foot anchoring. As they become more proficient with the drill, challenge them to slowly create more back leg flexion over time. Before implementing this drill, we highly recommend watching our Step-Back Throws video on our website to put a visual together with the drill explanation.
