# Supplementary material for: Effects of a six-week weighted-implement throwing program on baseball pitching velocity, kinematics, arm stress, and arm range of motion
Source: PeerJ. 2018 Nov 23;6:e6003. doi: 10.7717/peerj.6003 (PMC6254244; doi:10.7717/peerj.6003)
Supplement: Supplemental Information 7 — Referenced as HTKC2 in the study, these are exercise guidelines. [file peerj-06-6003-s007.pdf]

8

POST-THROWING RECOVERY WORK

A comprehensive post-throwing recovery program is just as important as a pre-throwing warm-up phase. Whether the athlete has just finished pitching in a game, completed a Pulldown session, or has just completed a typical non-velocity testing workout day, the arm needs proper recovery to ensure an optimal-training effect and to reduce the chance of overuse injuries. A huge factor in velocity development is the athletes' ability to put forth their best effort on a regular basis, so the objective is to minimize the amount of bad training days. Simply put, any athlete that is not recovering is not consistently training at a high level. Athletes should stick around 30 minutes or so after their primary throwing workout to complete their post-throwing recovery work. This is an *absolute requirement* at our training facility.

Many of the post-throwing recovery exercises are the same as the pre-throwing warm-up exercises, but there are also a significant number of drills done to

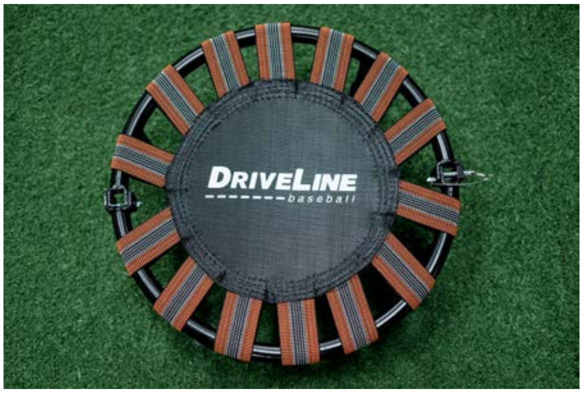

help reinforce existing patterns and develop force-acceptance proficiency as well as passive methods to ensure that tissue pliability stays high. Let's outline our core post-throwing recovery exercises that were not discussed in the Pre-Throwing Warm-up chapter, as well as a few alternate exercises for scenarios in which the required equipment for the core post-throwing recovery exercises is unavailable.

Standing Rebounders

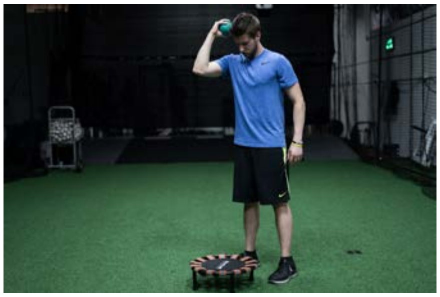

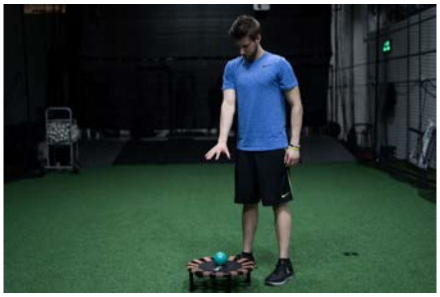

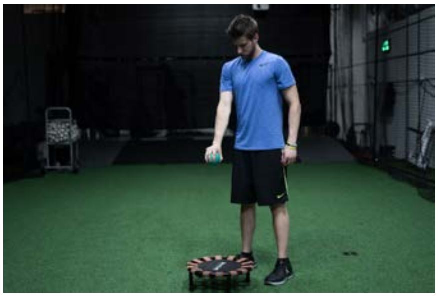

OVERVIEW

Catching PlyoCare balls that have been thrown against a mini-trampoline helps develop force acceptance at the elbow and shoulder, allowing those areas to absorb more stress during the pitching delivery by building a bigger deceleration engine. This is also a low-stress method to train the unwinding transition from supination to pronation and for athletes to feel how the ball is released from an ultimately neutral hand position. The specific weights of the PlyoCare balls used for this exercise can vary from program to program, but the goal is to use as heavy a PlyoCare ball as possible.

METHOD

With a PlyoCare ball in the throwing hand, stand close to the mini-trampoline. Making sure the athlete is close to the mini-trampoline is vital to train the correct unwinding of the arm, so one tip is to have the athlete place the feet on each side of one of the mini-trampoline legs. Once the starting position has been established, the next step is to raise the throwing arm up into a similar position as the Two-Arm Throws wrist-weights exercise (elbow up, palm next to the ear). The next step is to unwind the arm in a downward direction, throwing the ball into the trampoline. Here are two common mistakes to avoid while doing so:

1. *Pushing the ball*
2. *Unwinding with a supinated hand, similar to using a yo-yo*

The throwing arm should unwind so that it extends at waist height or below. Keep the arm extended, and catch the PlyoCare ball on the rebound, resisting the force pushing the arm upwards. If done correctly, the throwing arm will unwind and the ball will bounce off of the mini-trampoline back into the hand without having to move it.

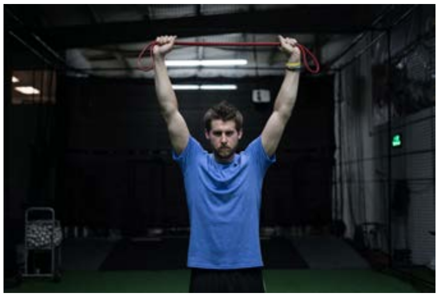

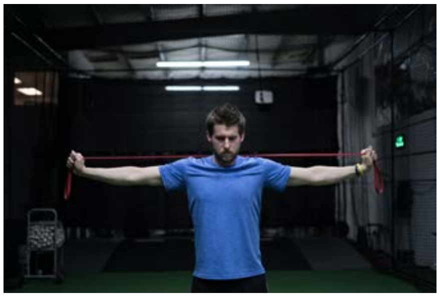

BAND PULLAPARTS SERIES:
Reverse Scap Pullaparts

OVERVIEW

This exercise helps cue and restore proper function of the shoulder blades by driving the scaps down and back during the movement.

METHOD

With a red Rogue Monster band or a set of J-Bands, have the athlete stand in a stationary position with the bands held in the hands over the head. Simply pull the band down and back across the top of the upper back. The athlete should focus on driving the elbows down and tucking them back. This exercise is often done for 10 reps per set.

BAND PULLAPARTS SERIES:
Anterior Band Pullaparts

OVERVIEW

This exercise helps drive the shoulders backwards and reverses the "rolled forward" posture many pitchers find themselves in. This activates the entire shoulder-scapula complex.

METHOD

To start, the athlete should stand in a stationary position and hold the band in front of the body with the palms facing downwards. While keeping the arms straight, the next step is to pull the band apart, which should cause it to touch the athlete's chest. Make sure to not lose tension at any point during or between reps. This exercise is often done on both 45-degree diagonal planes and the horizontal plane for 10 reps each (30 total).

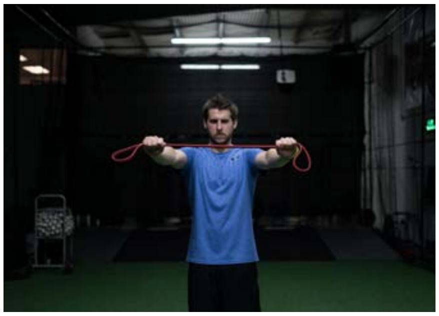

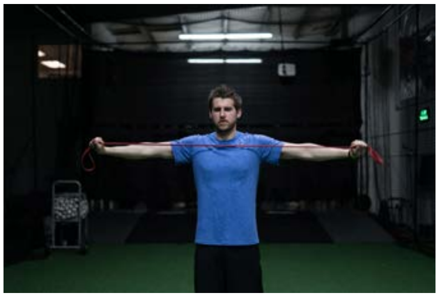

BAND PULLAPARTS SERIES:
No Money Drill

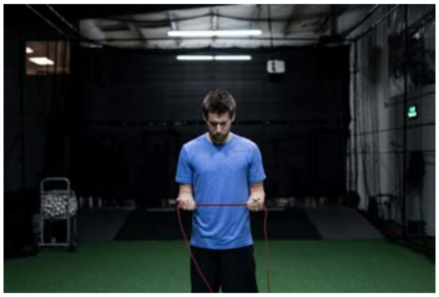

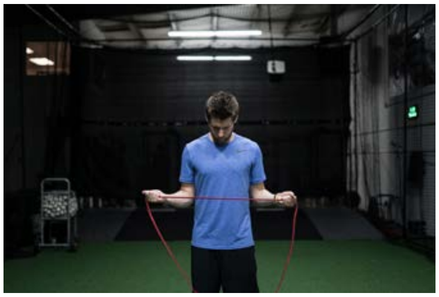

OVERVIEW

This exercise activates the scapula retractors and helps to depress the height of the humerus to open the subacromial space. This helps strengthen the external rotators and the infraspinatus.

METHOD

In a stationary standing position, have the athlete hold the band in front of them with the palms up and the elbows tucked in to their sides. Spread the band while externally rotating at the shoulder. *Do not* move the elbows away from the body. This is not meant to be a huge movement—quality of movement is far more important than distance traveled. This exercise is also often done for 10 reps per set.

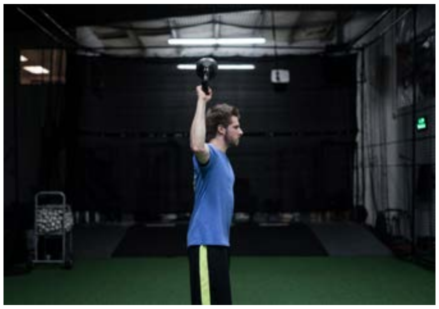

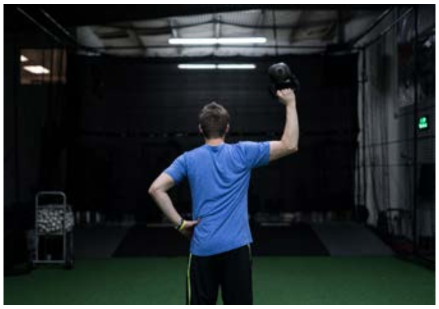

Waiter Walks

OVERVIEW

Waiter Walks challenge the dynamic stability of the shoulder-scapula complex as the rotator cuff fires on all cylinders to keep the weight in the air.

METHOD

The athlete should use a bottoms-up kettlebell grip (a weight plate is a decent substitute if no kettlebells are available) and hold the elbow directly to the side of the body at shoulder height. The wrist should be in a strong, fixed position—don't allow it to externally rotate, which will compromise the ability to hold the weight in the air. The free hand should hang down loosely. Once the starting position has been established, walk a defined distance (usually about 15-20 yards each direction) and keep the weight balanced in the waiter position. The athlete should try to keep the shoulders as level as possible while walking. If the weight happens to slip and fall, stop walking and reset the grip before proceeding with the walk towards the finish line.

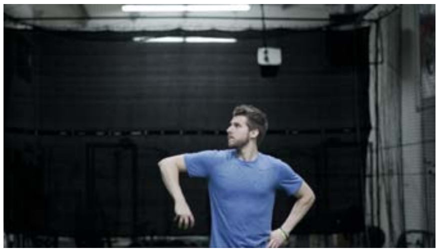

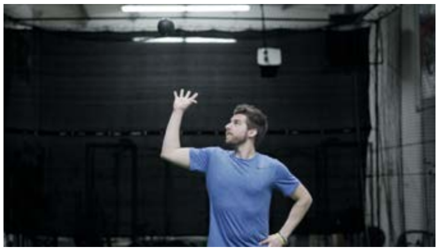

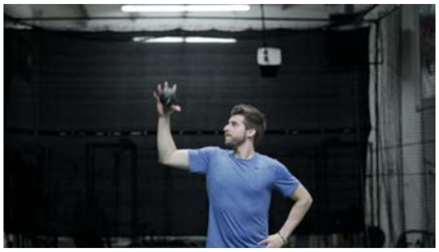

Standing Upward Tosses

OVERVIEW

Standing Upward Tosses help train both external-rotation strength and internal-rotation deceleration by externally rotating to throw the ball upwards into the air and internally rotating to catch the ball as it makes its way down to the ground. This also gives the athlete good kinesthetic feedback on how the shoulder should operate during the arm-cocking phase of the delivery—with minimal elbow movement during external rotation and loading. This exercise is usually done with the heaviest PlyoCare ball, if possible, and can serve as an adequate replacement for Standing Rebounders if a mini-trampoline is not available.

METHOD

Start out in a stationary standing position with the glove hand lightly braced against the chest. With the PlyoCare ball in the throwing hand, hold the throwing arm out to the side in an internally rotated position with the elbow at roughly shoulder height. Once the starting position has been established, have the athlete externally rotate the shoulder while keeping the elbow at the same height and throw the PlyoCare ball into the air. When the PlyoCare ball falls towards the ground, catch it while internally rotating at the shoulder, attempting to slowly decelerate the ball's flight. Deceleration should take approximately one second.

Side-Lying External Rotation Tosses

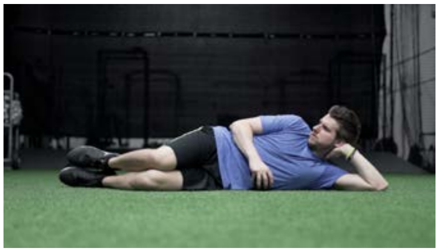

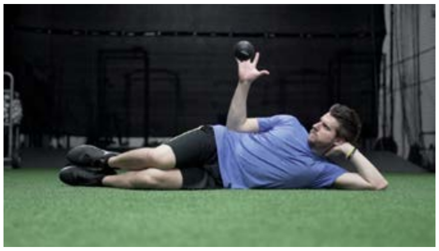

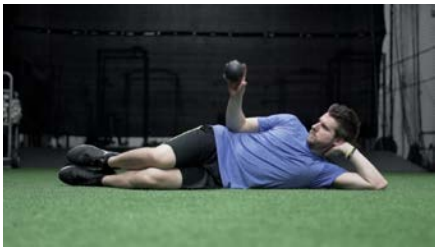

OVERVIEW

This exercise maximizes activity in the external rotators due to the constrained starting position. Like Standing Upward Tosses, this exercise is usually done with the heaviest PlyoCare ball, if possible, and can serve as an adequate replacement for Standing Rebounders if a mini-trampoline is not available.

METHOD

To start, have the athlete lie down on the glove side ribcage with a foam roller or other object propping the head up. Once in the starting position, hold the throwing-arm elbow against the side and externally rotate the shoulder to throw the ball up, repeating the same steps as the Standing Upward Tosses to catch and decelerate the ball when it come back down.
